# Dietary n-3 polyunsaturated fatty acids alter the number, fatty acid profile and coagulatory activity of circulating and platelet-derived extracellular vesicles: a randomized, controlled crossover trial

**DOI:** 10.1016/j.ajcnut.2024.03.008

**Published:** 2024-03-13

**Authors:** Esra Bozbas, Ruihan Zhou, Shin Soyama, Keith Allen-Redpath, Joanne L Mitchell, Helena L Fisk, Philip C Calder, Chris Jones, Jonathan M Gibbins, Roman Fischer, Svenja Hester, Parveen Yaqoob

**Affiliations:** 1 Hugh Sinclair Unit of Human Nutrition, Department of Food and Nutritional Sciences, University of Reading, Reading, United Kingdom; 2 Institute for Cardiovascular and Metabolic Research and School of Biological Sciences, University of Reading, Reading, United Kingdom; 3 School of Human Development and Health, Faculty of Medicine, University of Southampton, Southampton, United Kingdom; 4 NIHR Southampton Biomedical Research Centre, University Hospital Southampton NHS Foundation Trust and University of Southampton, Southampton, United Kingdom; 5 Target Discovery Institute, Centre for Medicines Discovery, Nuffield Department of Medicine, University of Oxford, Oxford, United Kingdom

**Keywords:** cardiovascular disease, extracellular vesicles, platelet-derived extracellular vesicles, fish oil, thrombosis, coagulation

## Abstract

**Background:**

Extracellular vesicles (EVs) are proposed to play a role in the development of cardiovascular diseases (CVDs) and are considered emerging markers of CVDs. n-3 PUFAs are abundant in oily fish and fish oil and are reported to reduce CVD risk, but there has been little research to date examining the effects of n-3 PUFAs on the generation and function of EVs.

**Objectives:**

We aimed to investigate the effects of fish oil supplementation on the number, generation, and function of EVs in subjects with moderate risk of CVDs.

**Methods:**

A total of 40 participants with moderate risk of CVDs were supplemented with capsules containing either fish oil (1.9 g/d n-3 PUFAs) or control oil (high-oleic safflower oil) for 12 wk in a randomized, double-blind, placebo-controlled crossover intervention study. The effects of fish oil supplementation on conventional CVD and thrombogenic risk markers were measured, along with the number and fatty acid composition of circulating and platelet-derived EVs (PDEVs). PDEV proteome profiles were evaluated, and their impact on coagulation was assessed using assays including fibrin clot formation, thrombin generation, fibrinolysis, and ex vivo thrombus formation.

**Results:**

n-3 PUFAs decreased the numbers of circulating EVs by 27%, doubled their n-3 PUFA content, and reduced their capacity to support thrombin generation by >20% in subjects at moderate risk of CVDs. EVs derived from n-3 PUFA-enriched platelets in vitro also resulted in lower thrombin generation, but did not alter thrombus formation in a whole blood ex vivo assay.

**Conclusions:**

Dietary n-3 PUFAs alter the number, composition, and function of EVs, reducing their coagulatory activity. This study provides clear evidence that EVs support thrombin generation and that this EV-dependent thrombin generation is reduced by n-3 PUFAs, which has implications for prevention and treatment of thrombosis.

**Clinical Trial Registry:**

This trial was registered at clinicaltrials.gov as NCT03203512.

## Introduction

Extracellular vesicles (EVs) are lipid bilayer-enclosed vesicles derived from almost all cells under both physiological and pathological conditions. These structures are reported to play roles in endothelial dysfunction, inflammation, and thrombosis [1], and there are positive associations between elevated circulating EV numbers and cardiovascular and thrombogenic risk markers [2], as well as hypercholesterolemia [3], dyslipidemia [4], and hypertension [5]. Numbers of EVs are increased in cardiovascular diseases (CVDs), including ischemic stroke [6] and coronary artery disease [7].

Platelet-derived EVs (PDEVs) comprise the major EV population in the circulation, representing 60%–90% of circulating EVs [8]. They are considered particularly important contributors to the development and progression of CVDs, potentially serving as biomarkers for cardiovascular health [9]. EVs released from platelets support platelet activation, thrombin generation, and thrombus formation [10], potentially playing an important role in vascular dysfunction and thrombosis [11]. Their coagulatory activity is attributed to exposure of negatively charged phospholipids, including phosphatidylserine (PS), which provide a catalytic surface for the assembly and activation of tenase (factors IXa, VIIIa, and X) and prothrombinase complexes (factors Xa, Va, and prothrombin), thereby supporting further activation of the coagulation cascade to generate thrombin and the subsequent conversion of fibrinogen to fibrin [12]. Indeed, EVs released from platelets are reported to be 50- to 100-fold more procoagulant than activated platelets because of elevated expression of PS, P-selectin, and factor X [13].

Dietary and lifestyle interventions have been demonstrated to modify CVD risk factors [14]. The cardioprotective effects of fish oil-derived n-3 PUFAs could be attributed to lowering of plasma triacylglycerol (TAG) concentration [15] and/or anti-inflammatory, antithrombogenic, and antiatherogenic properties [16]. Moreover, it is well-established that dietary n-3 PUFAs are incorporated into plasma phospholipids, platelets, and cell membranes [17]. Therefore, there is a sound basis for proposing that modulation of platelet and cell membranes by n-3 PUFAs could influence the generation, composition, and function of EVs. Although a few studies have investigated the effects of n-3 PUFA supplementation on EVs [[18], [19], [20], [21], [22], [23], [24], [25], [26], [27], [28]], the reported results are inconsistent. There are limitations in study design and methodology, and there is no insight into the impact of supplementation with n-3 PUFAs on the ability of platelets to generate EVs in vitro, nor is there any information about the characteristics and functional activities of EVs generated from n-3 PUFA-enriched platelets in vitro.

This randomized, double-blind, placebo-controlled, crossover trial aimed to investigate whether daily supplementation of participants at moderate risk of CVDs with 1.9 g/d of fish oil-derived n-3 PUFAs altered the generation, composition, and function of circulating and PDEVs.

## Methods

This study was conducted in the Hugh Sinclair Unit of Human Nutrition, School of Chemistry, Food and Pharmacy, University of Reading, following guidelines detailed in the Declaration of Helsinki and approved by the University of Reading Research Ethics Committee (reference: UREC 17/18). Written informed consent was obtained from participants.

### Trial design

The trial was a randomized, double-blind, placebo-controlled crossover. Eligible participants were randomly allocated to supplementation with capsules containing either fish oil or high-oleic safflower oil (control) in the first 12-wk treatment period, followed by a 12-wk washout period and then crossover to the other intervention for a further 12 wk. Random assignment of subjects for intervention order (“1” and “2”) was performed using online software (https://www.randomizer.org/). The study capsules were blinded by a researcher not involved in the stud,y and the code was not revealed until all statistical analyses had been completed. Anthropometric measurements (weight and height), blood pressure, and blood sampling were conducted at the beginning and end of each treatment period after an overnight fast, as previously described [2]. Participants were asked to abstain from alcohol and strenuous exercise (> 20 min 3 times/wk) during the 24 h prior to the study day and to continue with their normal diet, consuming no more than 2 portions of oily fish per month. Prior to enrolling in the study and on each visit day, participants returned completed food frequency questionnaires (FFQs), which were modified from the European Prospective Investigation of Cancer (EPIC)-Norfolk FFQ to include additional focus on the daily consumption of oily fish [29]. Data were analyzed by the FFQ EPIC Tool for Analysis (FETA) software and were used to record habitual dietary intakes during the previous year and dietary intake during the intervention periods, and the baseline data suggested that participants were consuming <1 portion of oily fish per month, below the required threshold of 2 portions/month. After the first visit, participants were asked to take 6 capsules/da of either fish oil, providing a total daily intake of 1.9 g n-3 PUFA (1080 mg EPA and 810 mg DHA) or high-oleic safflower oil, providing 740 mg oleic acid plus 120 mg linoleic acid, for 12 wks. The fish oil, an AlaskOmega omega-3 concentrate (EE 400300) derived from Alaskan Pollock (Gadus chalcogrammus), and safflower oil capsules were provided by Wiley Companies, Coshocton, Ohio. Participants were advised to take capsules with breakfast, lunch, and dinner (2 at each meal).

Compliance was monitored by providing participants with a daily checklist, which they returned at the end of each arm, by capsule counts, and by modification of the plasma fatty acid composition. Participants were provided with capsules in excess of requirements, and remaining capsules at the end of the 12-wk treatment period were counted. Compliance was >98% throughout the trial, as judged by capsule counts. Changes in the fatty acid composition of plasma total phospholipids also reflected good compliance, as indicated in the Results section.

### Participants

A total of 40 participants aged between 40 and 70 y (median 64 y) with moderate CVD risk, comprising 24 males and 16 females, recruited from the community of Reading, UK, completed the study, as illustrated in the participant flow diagram (Supplemental Figure 1). Out of 58 volunteers screened, 42 were enrolled in the trial between January 2018 and March 2019. Forty subjects successfully completed the trial in November 2019 with no serious adverse events and supplements well tolerated by all subjects.

Volunteers interested in the study were first assessed using a medical and lifestyle questionnaire administered via email or telephone. Potential eligible volunteers were invited to a screening visit to undergo anthropometric measurements and biochemical tests to assess their eligibility for the study. Moderate CVD risk was determined by the QRISK2 scoring system (https://qrisk.org/2017/), which is defined as 10%–20% risk of a heart attack or stroke in the next 10 y. QRISK2 is based on 9 risk factors, including age, systolic blood pressure, smoking status, ratio of total serum cholesterol to high-density lipoprotein cholesterol), BMI (kg/m^2^), ethnicity, measures of deprivation, and family history (CVD in first degree relative <60 y). Exclusion criteria were underweight (<18.5 kg/m^2^); anemia (hemoglobin concentration ＜12.5 g/L in males and ＜11.5 g/L in females); hyperlipidemia (total cholesterol >8 mmol/L); diabetes (diagnozed or fasting glucose concentration ＞7 mmol/L) or other endocrine disorders; angina, stroke, or any vascular disease in the past 12 months; renal, gastrointestinal, respiratory, liver or bowel disease; inflammatory disease; drug treatment for hypertension, hyperlipidemia, inflammation, depression or thyropathy; aspirin, ibuprofen or other nonsteroidal anti-inflammatory drugs (NSAIDs) >4 times per month, or once in the week preceding the study; any other antiplatelet or anticoagulant medication (e.g., triflusal, clopidogrel, or warfarin); allergies; smoking (including e-cigarettes and nicotine products); alcohol misuse or intakes ＞21 units/wk for males and ＞15 units/wk for females or a history of alcohol misuse; regular consumption of oily fish (>2 portions/month); consumption of dietary supplements; planning to start or on a weight reducing regimen; intense aerobic exercise (＞20 min, 3 times/wk); pregnancy or lactation, or if of reproductive age and not using a reliable form of contraception (including abstinence); participation in another clinical trial within the last 3 months. All enrolled participants were nonsmokers, without any diagnosed diseases, and not receiving any medication and/or supplements.

Baseline characteristics of the 40 participants completing the study were reported, and associations between these characteristics and EV numbers and function have been previously published [2]. The study population was mildly hypertensive (SBP = 134 ± 2.2 mmHg) and mildly hypercholesterolemic (total cholesterol = 6 mmol/L).

### Sample size

A sample size calculation was performed for the main endpoints: EV numbers, thrombus formation, and platelet aggregation. Based on a previous study [22], a total of 34 participants was considered sufficient to detect a 10% reduction in the number of EVs following fish oil supplementation, with a 2-sided significance level of 5% and a power of 95%. A recruitment target of 40 allowed for a 15% dropout.

### Blood collection and processing

Venous blood samples were collected into 3.2% sodium citrate tubes and processed as previously described [2]. Platelet-rich plasma (PRP) and platelet-poor plasma (PPP) were used in platelet aggregation, whereas the remaining PRP was used to isolate platelets. Platelet-free plasma (PFP) was used for the enumeration and characterization of circulating EVs or stored at −80 °C for the further analysis of thrombogenic activity of EVs (thrombin generation) and of PFP (thrombin generation and fibrin clot properties).

### Measurement of platelet aggregation

A high throughput, plate-based, platelet aggregation was conducted as previously described [2].

### Thrombodynamics analysis for fibrin clot formation and fibrinolysis

Thrombodynamics analysis was performed to determine the formation of a fibrin clot using a thrombodynamics analyzer and thrombodynamics kit (HemaCore), as described by Zhou et al. [2].

### Isolation of EVs

Circulating EVs from PFP were isolated by size exclusion chromatography using Izon qEV columns (Izon Science Ltd). Fractions 7–9 were pooled together and concentrated using a centrifugal concentrator (Fisher Scientific) at 1000 × g for 10 min at room temperature.

The isolation process for PDEVs generated in vitro from unstimulated and stimulated platelets consisted of i) isolation of platelets from whole blood, ii) stimulation (or not) of isolated platelets with thrombin receptor activator peptide-6 (TRAP-6), and iii) isolation of EVs from the supernatants of stimulated and unstimulated platelets as previously described [30]. In brief, isolated platelets at 3 × 108 platelets/mL were stimulated with either 30 μM of TRAP-6 (stimulated platelets) or PBS (unstimulated platelets) in the presence of 2 mM of CaCl_2_ (Sigma-Aldrich) and incubated at 37°C for 2 h. Platelets were then removed by 2 sequential centrifugations at 1200 × g for 10 min. The upper 90% of the supernatant, containing the EVs, was collected and pelleted by centrifugation at 15,000 × g for 30 min at 4°C. The supernatant was discarded, and the pellet rich in PDEVs was resuspended. Isolated PDEVs were pooled and stored in aliquots at −80ºC until use.

### Enumeration and characterization of EVs

#### Nanoparticle tracking analysis

The size distribution and concentration of EVs were determined by Nanoparticle tracking analysis (NTA) using a NanoSight 300 (NS300; Malvern) [2].

#### Characterization of EVs using flow cytometry

A flow cytometer (FCM; Canto II Flow Cytometer, BD Biosciences, UK), equipped with a blue (488 nm), red (633 nm), and violet (405 nm) laser, was used to characterize circulating EV subpopulations and detect PS expression on PDEVs generated in vitro from platelets, details of which have been previously reported in Zhou et al. [2] and Ferreira et al. [30] respectively.

#### Measurement of protein concentration of EVs using NanoDrop

Protein concentration measurements were conducted by a NanoDrop-1000 spectrophotometer using protein A280 measurements following the manufacturer’s instruction (Thermo Scientific). Prior to measurement, the instrument was cleaned with distilled water and blanked using 2 μL of nuclease20-free water. Sample (2 μL) was then loaded onto the pedestal, and this was repeated 3 times to obtain average protein concentration.

### Preparation of pooled vesicle-depleted plasma

Venous blood samples were collected from 3 healthy, fasted participants (not involved in the trial) to prepare pooled vesicle-depleted plasma (VDP). Whole blood was first centrifuged twice at 2500 × g for 15 min to remove blood cells and obtain pooled plasma. Vesicles were depleted from the pooled plasma by ultracentrifugation at 20,000 × g for 1 h at 4 °C, which pellets the EVs, leaving vesicle-poor supernatant. The vesicle-poor supernatant was ultracentrifuged at 100,000 × g for 1 h at 4 °C, followed by filtration 4 times through a 0.1 μm filter (Merk Millipore). This filtered supernatant was considered VDP and stored in 1 mL aliquots at −80 °C until use. Removal of vesicles at each stage of preparation of VDP was verified by NTA and FCM (Annexin V staining), which showed that 86.4% of particles detectable by NTA were removed from plasma, with 97.3% removal of PS+ EVs from plasma, as shown in Supplemental Figure 2.

### Functional assays of PDEVs

The effect of fish oil on EV functions was assessed through different stages of the coagulation cascade in functional assays, including clot formation, thrombin generation, fibrinolysis and thrombus formation. Full details of functional assays are provided in the Supplemental Methods.

#### Measurement of thrombin generation

Thrombin formation was assessed using a commercially available, plate-based thrombin generation assay (Technothrombin TGA kit), which assesses a change in fluorescence as a result of cleavage of a fluorogenic substrate by thrombin over time upon activation of the clotting cascade by tissue factor. Two separate analyses were conducted: (i) determination of the effect of n-3 PUFA supplementation on thrombin generation in PFP from study samples relative to pooled VDP and (ii) determination of the effects of circulating EVs and in vitro-generated PDEVs derived from study samples on thrombin generation in pooled VDP. Prior to analysis, a thrombin calibration curve was constructed from dilutions of lyophilized Hepes-NaCl-buffer containing 0.5 % bovine serum albumin and ∼1000 nM thrombin in buffer with BSA. A kinetic reading of the plate was initiated by addition of 50 μL of fluorogenic substrate solution containing fluorogenic substrate 1 mM Z-G-G-R-AMC and 15 mM CaCl2. Calibration curves were recorded at 37 °C for 10 min with 30 s intervals using the plate reader (FlexStation 3) at 360 nm for excitation and at 460 nm for emission.

The methods for both approaches were based on the use of pooled VDP as a negative control to allow assessment of thrombin generation specifically resulting from the presence of EVs. For the first approach, 40 μL aliquots of either prethawed study sample PFP or pooled VDP or pooled PFP were added to the plate. For the second approach, PDEVs (10 μL of EV suspensions at 5 μg/mL final protein concentration) produced from either unstimulated or stimulated platelets (UP-EVs and SP-EVs, respectively) from intervention samples or PBS (negative control) were added to 30 μL VDP. The concentration of 5 μg/mL protein was determined through trials as an appropriate concentration for thrombin generation in this assay. This was followed by addition of a 10 μL suspension of phospholipid micelles containing recombinant human tissue factor (TF) in Tris-Hepes-NaCl buffer (RCL), which was provided in the kit. Formation of thrombin was initiated by addition of 50 μL of fluorogenic substrate solution containing 1 mM Z-G-G-R-AMC and 15 mM CaCl2. Plates were immediately read at 37 °C for 1 h at 1 min intervals using a fluorescence plate reader (FlexStation 3) at excitation and emission wavelengths of 360 and 460 nm, respectively. All samples were measured in duplicate. Fluorescence intensity was detected by TGA Evaluation Software to calculate thrombin generation in samples. Data were then analyzed by the TGA Evaluation Software manually to convert the unit of thrombin generation from RFU to nM and presented as 5 variables: lag time, time to the peak, peak concentration of thrombin (nM), velocity index, and AUC.

### Composition of EVs

To determine the composition of EVs fatty acid analysis and proteomics were performed, as described in the Supplemental Methods.

### Statistical analysis

Dose-response curves of platelet aggregation for each baseline and intervention were constructed using a 4-parameter log-linear function in GraphPad Prism Software 9 (GraphPad). These dose-response curves of platelet aggregation were used to calculate relative parameters, including LogEC50, maximum response, minimum, and hill slope values. Comparisons of these parameters after each intervention were made using 2-way ANOVA with the Tukey multiple comparisons test. These statistical analyses were carried out using GraphPad Prism Software 9. Error bars denote SEM, and a *P* value of <0.05 was considered statistically significant.

Comparisons after each intervention for other assays were made using a General Linear Model (GLM), fitted to analyze time course data for study test points in order to determine individual treatment effects with fixed factors of time (repeated measures) and treatment. SPSS 24.0 software for Windows (SPSS, Inc.) was used to perform this statistical analysis. Error bars denote SEM, and a *P* value of <0.05 was considered statistically significant.

## Results

The study population exhibited mild hypertension (SBP = 134 ± 2.2) and mild hypercholesterolemia (total cholesterol = 6 mmol/L). Baseline characteristics of the 40 participants completing the study and associations between these characteristics and EV numbers and functions have been previously published; there was no influence of baseline characteristics on the outcomes of the trial [2]. FFQ analysis demonstrated that subjects consumed a diet containing ∼60–65 g/d total fat, of which <1.5 g/d was derived from n-3 PUFAs (Supplemental Table 1). There was no impact of the intervention on habitual dietary intakes (Supplemental Table 1).

### Supplementation with n-3 PUFAs altered the fatty acid profile of plasma phospholipids and lowered blood pressure and plasma TAG concentration

As expected, there was a broad range of effects of fish oil on plasma lipids, blood pressure, and plasma fatty acid composition, chiefly a lowering of plasma TAG concentration, an increase in plasma LDL-C concentration, a lowering of SBP, and increased proportions of n-3 PUFAs in plasma phosphatidylcholine and phosphatidylethanolamine. These effects provide confirmatory context for the trial and are described in Supplemental Tables 2–4.

### Supplementation with n-3 PUFAs decreased clot growth, clot size, and thrombin generation but did not alter fibrinolysis or platelet aggregation

Fish oil supplementation affected some aspects of coagulation: it decreased the rate of clot growth and clot size at 30 min but did not affect clot density, fibrinolysis parameters, or platelet aggregation in response to a range of agonists (Supplemental Figure 3; Supplemental Table 5).

### Supplementation with n-3 PUFAs decreased numbers of circulating EVs

**FIGURE 1:**
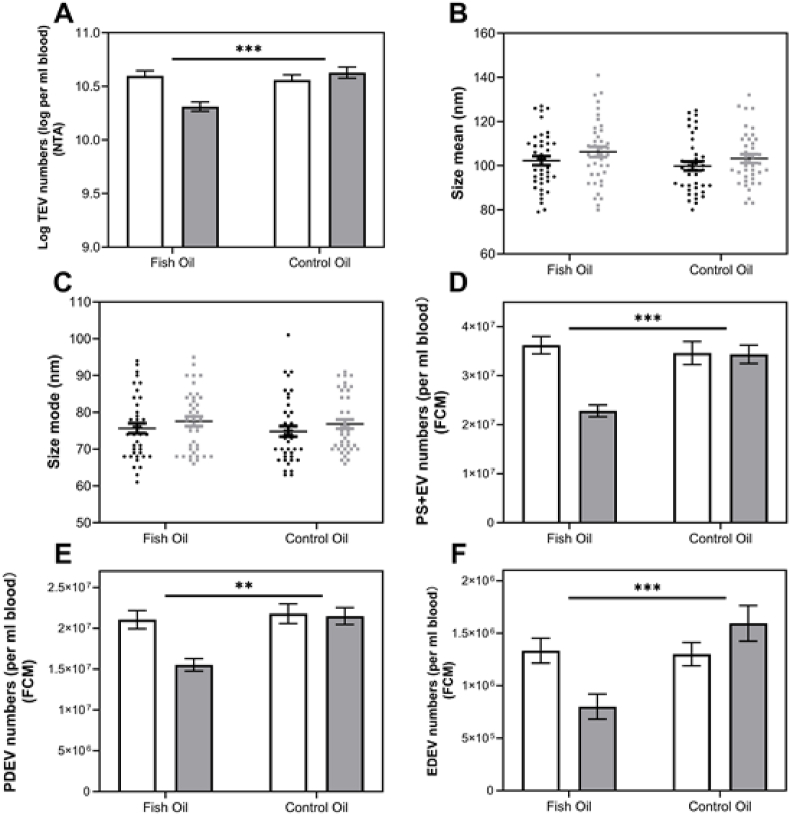
Numbers and size of circulating EVs before and after intervention. Data are mean ± SEM (n = 40). Data were analyzed using the General Linear Model (GLM), including pairwise comparison test with Bonferroni for treatment, period, and treatment∗time interaction with differences shown at *P* < 0.05. (A) Circulating EV numbers were significantly decreased in response to fish oil compared to control oil, but there were no effects on either (B) mean or (C) mode size. (D) PS+EV, (E) PS+PDEV and (F) EDEV numbers were significantly decreased following fish oil supplementation. NTA, nanoparticle tracking analysis; EV, extracellular vesicles.

Supplementation with fish oil significantly decreased numbers of circulating EVs (Figure 1A) but did not affect size or size distribution (Figure 1B, C). Notably, numbers of PS-positive circulating EVs, PDEVs, and endothelial cell-derived EVs (EDEVs) were decreased significantly by fish oil supplementation compared with the control oil (Figure 1D-F).

### Supplementation with n-3 PUFAs decreased EV-dependent thrombin generation

**FIGURE 2:**
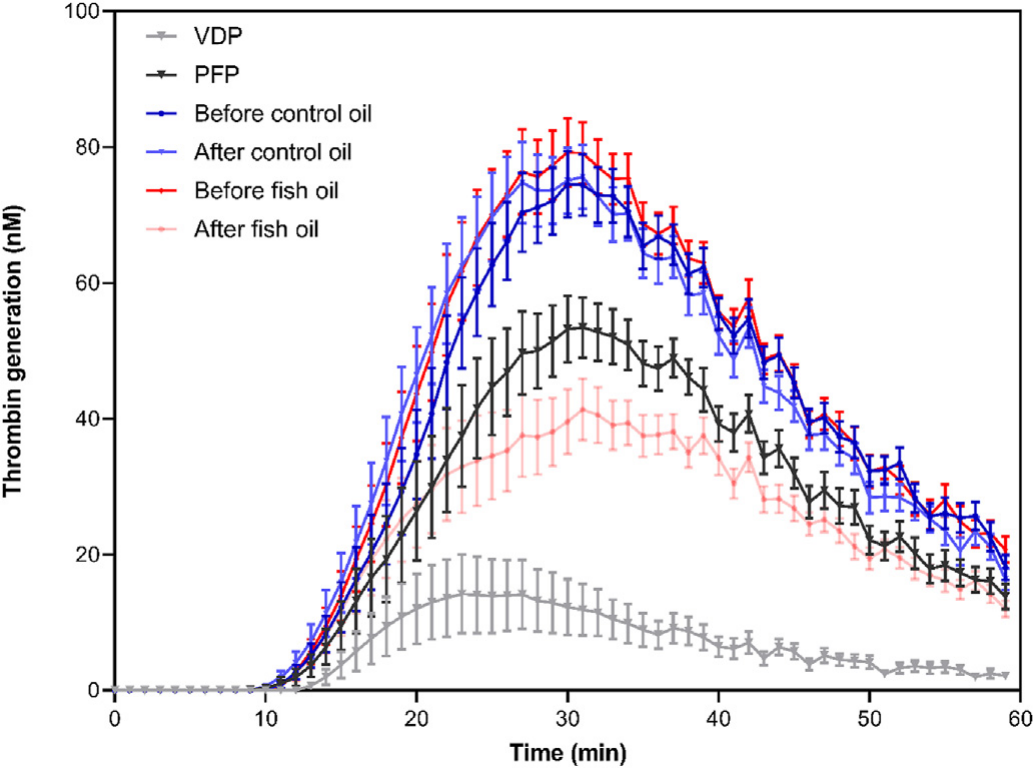
Effect of EVs and fish oil supplementation on thrombin generation over a 60-min time course. Pooled VDP and pooled PFP from 3 healthy individuals were used for benchmarking purposes in the assessment of thrombin generation in PFP from subjects participating in the intervention study. Thrombin-dependent cleavage of a fluorogenic substrate was quantified over 60 min. Data are mean ± SEM, (n = 3 for pooled VDP and PFP; n = 40 for the intervention). VDP, vesicle-depleted plasma; PFP, platelet-free plasma.

**FIGURE 3:**
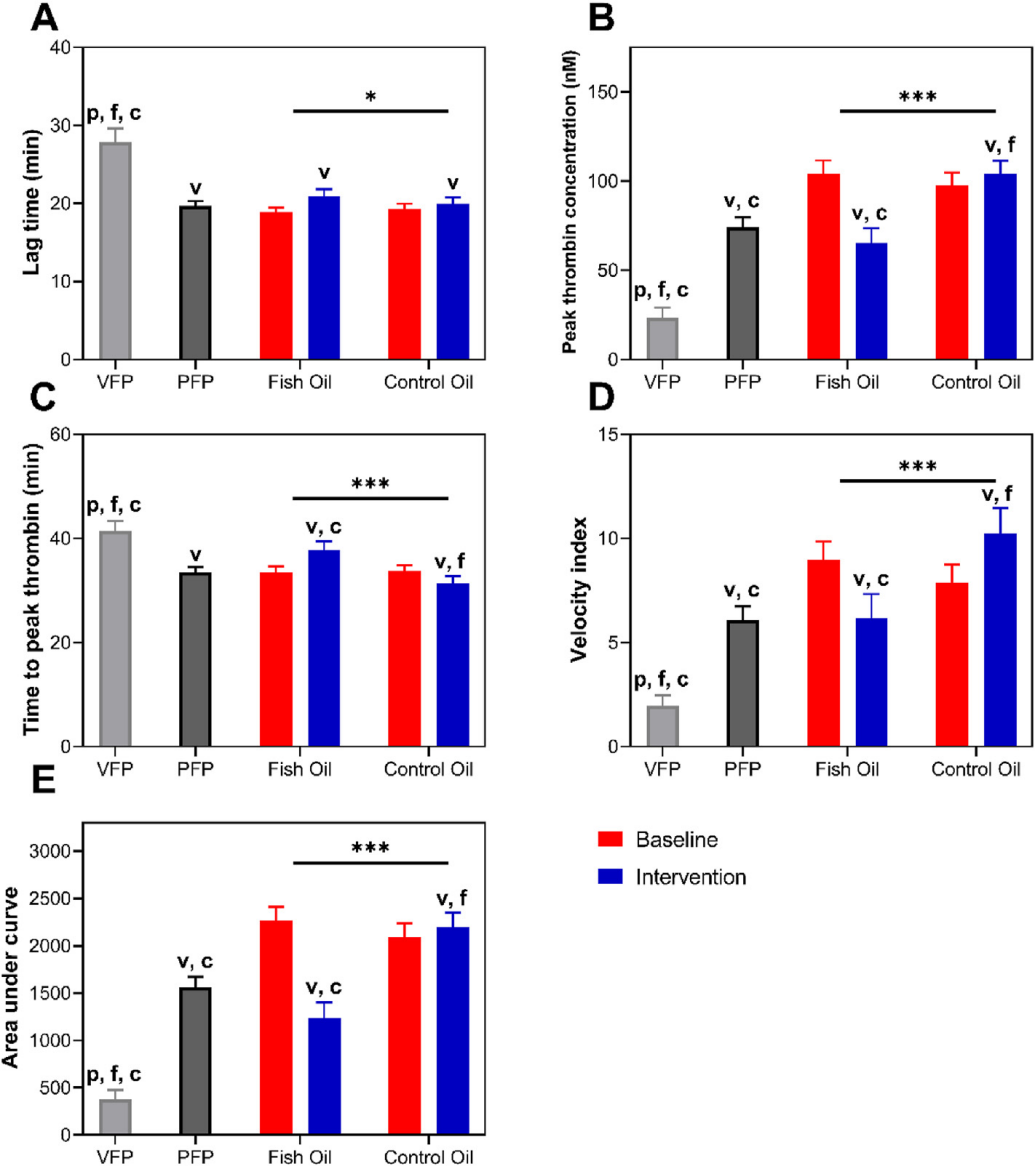
Thrombin generation parameters before and after intervention. Data are mean ± SEM (n = 40). Pooled VDP and pooled PFP were used for benchmarking purposes in the assessment of thrombin generation in PFP from participants in the intervention study. Data were analyzed using the General Linear Model (GLM), including pairwise comparison test with Bonferroni for treatment, period, and treatment∗time interaction with differences shown at *P* < 0.05. Comparisons of the means between PFP, VDP, after fish oil intervention and after control oil intervention were drawn using 1-way ANOVA, followed by the Tukey multiple comparison test, with differences shown as *P* < 0.05. The symbol of p denotes significantly different from PFP (*P* < 0.05); a symbol of **v** denotes significantly different from VDP (*P* < 0.05); a symbol of f denotes significantly different from fish oil postintervention (*P* < 0.05) and a symbol of **c** denotes significantly different from after control oil postintervention (*P* < 0.05). ∗*P* < 0.05 and ∗∗∗*P* < 0.001. VDP, vesicle-depleted plasma; PFP, platelet-free plasma.

Thrombin generation in pooled PFP from healthy individuals (n=3) was approximately double that in VDP, demonstrating that the presence of EVs in PFP supports EV-dependent thrombin generation (Figure 2). The absence of vesicles in VDP resulted in significantly prolonged lag time and time to reach peak thrombin generation, as well as lower peak thrombin concentration, slope (velocity index), and AUC compared with pooled PFP from the same participants (FIGURE 2, FIGURE 3).

Prior to supplementation, thrombin generation in pooled PFP from study participants tended to be higher than that in the pooled samples from healthy individuals (FIGURE 2, FIGURE 3). Supplementation with fish oil resulted in a reduction in peak thrombin generation, time to reach peak thrombin generation, velocity index and AUC, and prolonged lag time for thrombin generation (FIGURE 2, FIGURE 3) to the extent that at 30–40 min, thrombin generation in PFP from participants supplemented with fish oil was lower than that in the pooled PFP from healthy individuals (Figure 2). This indicates that overall, there was less thrombogenic activity in platelet-free plasma following fish oil supplementation, and the comparison with VDP suggests that this was at least partly attributable to EVs.

**FIGURE 4:**
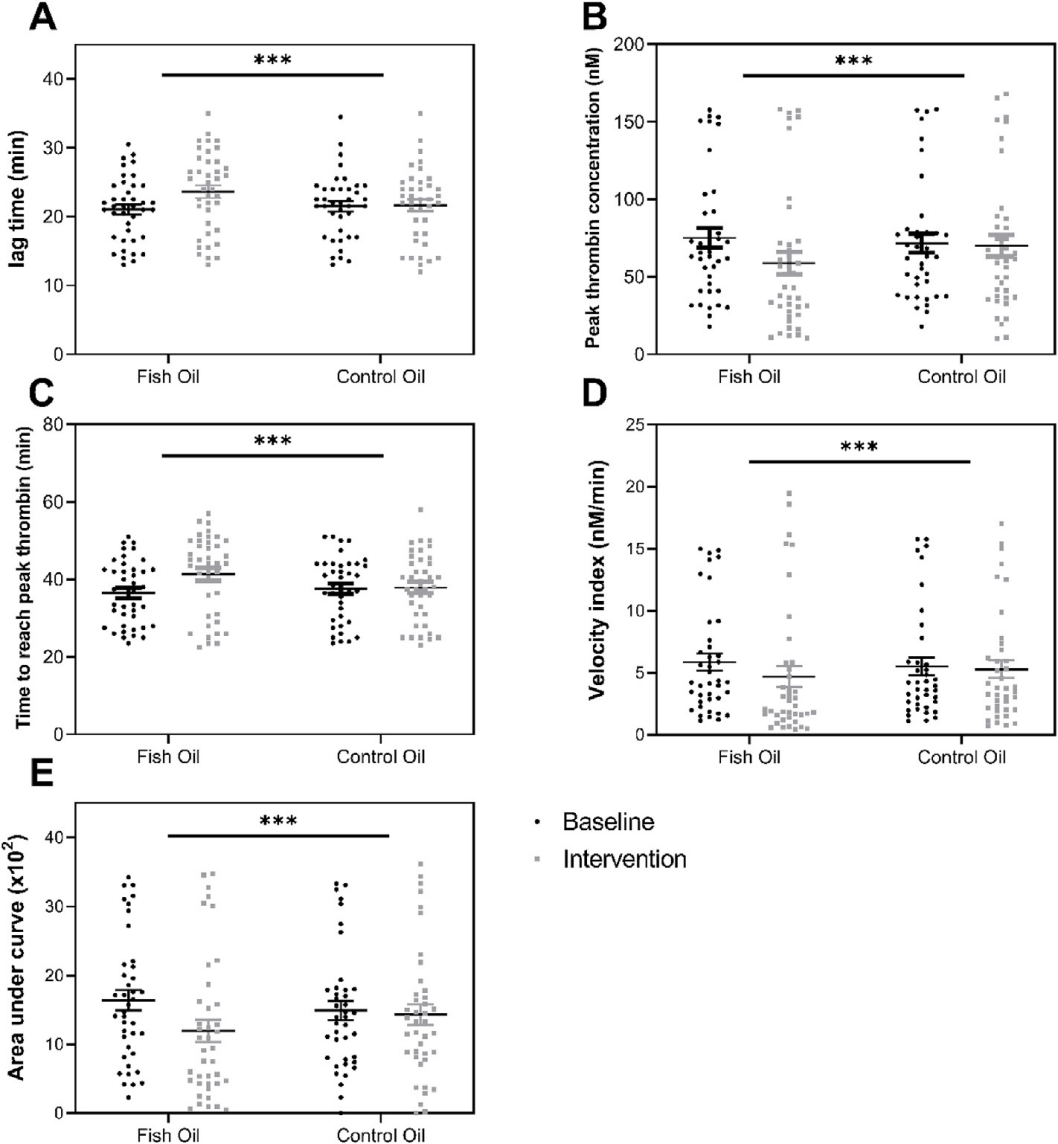
Capacity of EVs to support TF-dependent thrombin generation before and after intervention. Data are mean ± SEM, (n = 40). Comparisons after each intervention were drawn using the General Linear Model (GLM), including pairwise comparison with Bonferroni tests for treatment, period, and treatment∗time interaction, with differences shown at *P* < 0.05. Pooled VDP from healthy individuals (n = 3) was used as negative control. There was a significant effect of fish oil on (A) lag time for thrombin generation, (B) peak thrombin concentration, (C) time to reach peak thrombin, (D) velocity index, and (E) AUC for circulating EVs (treatment effects: *P* < 0.05; general linear model). *∗∗∗P* < 0.001. EVs, extracellular vesicles.

Figure 4 represents thrombin generation in VDP to which EVs from participants supplemented with fish oil were added. EVs modified by n-3 PUFAs were less able to support TF-dependent thrombin generation than those from participants supplemented with control oil, confirming that the effect of n-3 PUFAs on TF-dependent thrombin generation was mediated through EVs.

### Supplementation with n-3 PUFAs altered the fatty acid profile of circulating EVs and EVs generated in vitro from platelets

Fish oil supplementation did not alter the generation or size distribution of EVs from stimulated or unstimulated platelets in vitro, but it did decrease the expression of PS by PDEVs derived from unstimulated platelets (data not shown). n-3 PUFA supplementation more than doubled the content of EPA and DHA in circulating EVs and significantly increased the proportion of DPA, resulting in a substantial overall increase in total n-3 PUFAs. Supplementation also significantly decreased the proportions of oleic acid and arachidonic acid (AA) in circulating EVs (Table 1). There was a significant treatment∗time interaction for DPA (*P* = 0.017), suggesting that the effect of fish oil on DPA was time-dependent.

**TABLE 1:** Effect of fish oil supplementation on the fatty acid composition of circulating EVs

Variable	Fish Oil	After (wt%)	Control Oil	After (wt%)	*P* value
	Before (wt%)		Before (wt%)		treatment
Palmitic acid (16:0)	26.9 ± 0.5	26.8 ± 0.4	27.0 ± 0.6	26.6 ± 0.5	0.996
Stearic acid (18:0)	10.9 ± 0.6	11.4 ± 0.7	10.3 ± 0.6	10.3 ± 0.6	0.636
Oleic acid (18:1n-9)	29.4 ± 0.7	27.5 ± 0.7	29.9 ± 0.7	30.4 ± 0.8	0.011
Linoleic acid (18:2n-6)	17.1 ± 0.5	17.0 ± 0.6	17.7 ± 0.5	17.6 ± 0.5	0.807
ALA (18:3n-3)	1.1 ± 0.1	1.12 ± 0.1	1.1 ± 0.1	1.1 ± 0.1	0.461
DGLA (20:3n-6)	1.8 ± 0.1	1.7 ± 0.1	1.8 ± 0.1	1.7 ± 0.1	0.718
AA (20:4n-6)	2.9 ± 0.1	2.5 ± 0.1	2.9 ± 0.1	2.9 ± 0.1	<0.001
ETA (20:4n-3)	1.7 ± 0.2	2.0 ± 0.2	1.4 ± 0.2	1.5 ± 0.2	0.436
EPA (20:5n-3)	0.7 ± 0.1	1.6 ± 0.1	0.6 ± 0.1	0.5 ± 0.1	<0.001
DPA (22:5n-3)	0.4 ± 0.03	0.6 ± 0.03	0.4 ± 0.02	0.4 ± 0.03	0.004
DHA (22:6n-3)	0.9 ± 0.1	1.9 ± 0.1	0.9 ± 0.1	0.9 ± 0.1	<0.001
Total SFAs	37.8 ± 0.9	38.2 ± 0.9	37.3 ± 0.9	36.9 ± 0.9	0.773
Total MUFAs	34.3 ± 0.8	32.1 ± 0.8	34.9 ± 0.8	35.1 ± 0.9	0.013
Total n-3 PUFAs	4.7 ± 0.3	7.2 ± 0.2	4.3 ± 0.2	4.5 ± 0.2	<0.001
Total n-6 PUFAs	21.9 ± 0.6	21.2 ± 0.6	22.3 ± 0.6	22.2 ± 0.5	0.627

Data are mean ± SEM (n = 40). Comparisons after each intervention were drawn using General Linear Model (GLM), with differences shown at *P* < 0.05.

Abbreviations: AA, arachidonic acid; ALA, alpha-linolenic acid; DGLA, dihomo-γ-linolenic acid; ETA, eicosatetraenoic acid; EPA, eicosapentaenoic acid; DPA, docosapentaenoic acid; DHA, docosahexaenoic acid; MUFA, monounsaturated fatty acid; PUFA, polyunsaturated fatty acid; SFA, saturated fatty acid.

Intervention with fish oil significantly increased the n-3 PUFA content of PDEVs derived from both stimulated and unstimulated platelets in vitro, whereas decreasing that of AA. In stimulated platelets there also appeared to be enrichment of GLA, ALA, and eicosenoic acid following fish oil supplementation (Table 2).

**TABLE 2:** Effect of fish oil supplementation on the fatty acid composition of EVs derived from unstimulated or stimulated platelet.

Variable	EVs from unstimulated platelets					EVs from stimulated platelets				
	Fish Oil		Control Oil		*P* value	Fish Oil		Control Oil		*P* value
	Before (wt%)	After (wt%)	Before (wt%)	After (wt%)	treatment	Before (wt%)	After (wt%)	Before (wt%)	After (wt%)	treatment
Palmitic acid (16:0)	35.0 ± 1.0	34.9 ± 1.0	34.7 ± 0.9	34.3 ± 1.1	0.570	33.9 ± 1.1	33.7 ± 1.0	33.8 ± 0.9	34.8 ± 1.0	0.324
Stearic acid (18:0)	31.3 ± 0.8	31.3 ± 0.8	31.2 ± 0.8	31.0 ± 0.6	0.971	30.7 ± 0.8	31.6 ± 0.8	31.4 ± 0.7	30.5 ± 0.7	0.089
Oleic acid (18:1n-9)	7.9 ± 0.4	8.1 ± 0.5	7.8 ± 0.4	8.0 ± 0.4	0.686	9.1 ± 0.5	8.3 ± 0.5	8.6 ± 0.5	8.7 ± 0.5	0.236
Linoleic acid (18:2n-6)	2.6 ± 0.2	2.5 ± 0.2	2.5 ± 0.2	2.7 ± 0.2	0.433	2.8 ± 0.2	2.6 ± 0.2	2.6 ± 0.2	2.7 ± 0.2	0.196
GLA (18:3n-6)	1.0 ± 0.1	1.0 ± 0.1	0.9 ± 0.1	0.9 ± 0.1	0.350	0.9 ± 0.1	1.0 ± 0.1	1.0 ± 0.1	0.8 ± 0.1	0.006
ALA (18:3n-3)	2.8 ± 0.2	2.8 ± 0.2	2.9 ± 0.2	2.8 ± 0.3	0.236	2.7 ± 0.2	2.9 ± 0.3	2.9 ± 0.2	2.8 ± 0.3	0.017
Eicosenoic acid (20:1n-9)	1.9 ± 0.2	2.1 ± 0.1	1.9 ± 0.1	1.9 ± 0.1	0.291	1.9 ± 0.2	1.9 ± 0.1	2.1 ± 0.1	1.6 ± 0.1	0.011
DGLA (20:3n-6)	7.0 ± 0.4	7.3 ± 0.5	7.5 ± 0.5	7.6 ± 0.4	0.439	6.9 ± 0.5	7.1 ± 0.5	6.9 ± 0.4	7.0 ± 0.4	0.663
AA (20:4n-6)	4.0 ± 0.5	2.9 ± 0.3	3.7 ± 0.4	4.4 ± 0.5	0.021	5.1 ± 0.7	4.0 ± 0.6	4.0 ± 0.4	4.7 ± 0.6	0.011
ETA (20:4n-3)	0.4 ± 0.1	0.6 ± 0.1	0.4 ± 0.1	0.4 ± 0.1	0.006	0.4 ± 0.1	0.6 ± 0.0	0.4 ± 0.1	0.5 ± 0.1	0.167
EPA (20:5n-3)	0.8 ± 0.1	1.2 ± 0.2	1.0 ± 0.1	0.9 ± 0.1	0.018	0.6 ± 0.1	1.0 ± 0.1	0.9 ± 0.1	0.8 ± 0.1	0.042
DPA (22:5n-3)	0.6 ± 0.1	0.6 ± 0.1	0.6 ± 0.1	0.6 ± 0.1	0.676	0.7 ± 0.1	0.7 ± 0.1	0.6 ± 0.1	0.6 ± 0.1	0.689
DHA (22:6n-3)	0.4 ± 0.0	0.5 ± 0.0	0.4 ± 0.0	0.5 ± 0.0	0.863	0.4 ± 0.0	0.5 ± 0.1	0.4 ± 0.0	0.4 ± 0.0	0.282
Total SFAs	68.0 ± 1.0	67.9 ± 0.9	67.7 ± 1.8	67.0 ± 1.1	0.600	66.2 ± 1.0	67.0 ± 1.0	66.9 ± 1.0	67.0 ± 1.0	0.692
Total MUFAs	11.6 ± 0.5	11.7 ± 0.5	11.3 ± 0.7	11.4 ± 0.5	0.761	12.4 ± 0.5	11.7 ± 0.5	12.4 ± 0.6	11.9 ± 0.5	0.759
Total n-3 PUFAs	5.0 ± 0.3	5.8 ± 0.3	5.3 ± 0.6	5.1 ± 0.3	0.010	4.9 ± 0.3	5.7 ± 0.4	5.2 ± 0.4	5.0 ± 0.3	0.003
Total n-6 PUFAs	15.4 ± 0.6	14.6 ± 0.5	15.7 ± 1.2	16.5 ± 0.6	0.069	16.5 ± 0.7	15.5 ± 0.6	15.5 ± 0.6	16.2 ± 0.6	0.114

Data are mean ± SEM (n = 40). Comparisons after each intervention were drawn using General Linear Model (GLM), with differences shown at *P* < 0.05.

Abbreviations: AA, arachidonic acid; ALA, alpha-linolenic acid; DGLA, dihomo-γ-linolenic acid; ETA, eicosatetraenoic acid; EPA, eicosapentaenoic acid; DPA, docosapentaenoic acid; DHA, docosahexaenoic acid; GLA, gamma linolenic acid; MUFA, monounsaturated fatty acid; PUFA, polyunsaturated fatty acid; SFA, saturated fatty acid.

### PDEVs from platelets enriched with n-3 PUFAs altered clot formation and thrombin generation but not ex vivo thrombus formation

**FIGURE 5:**
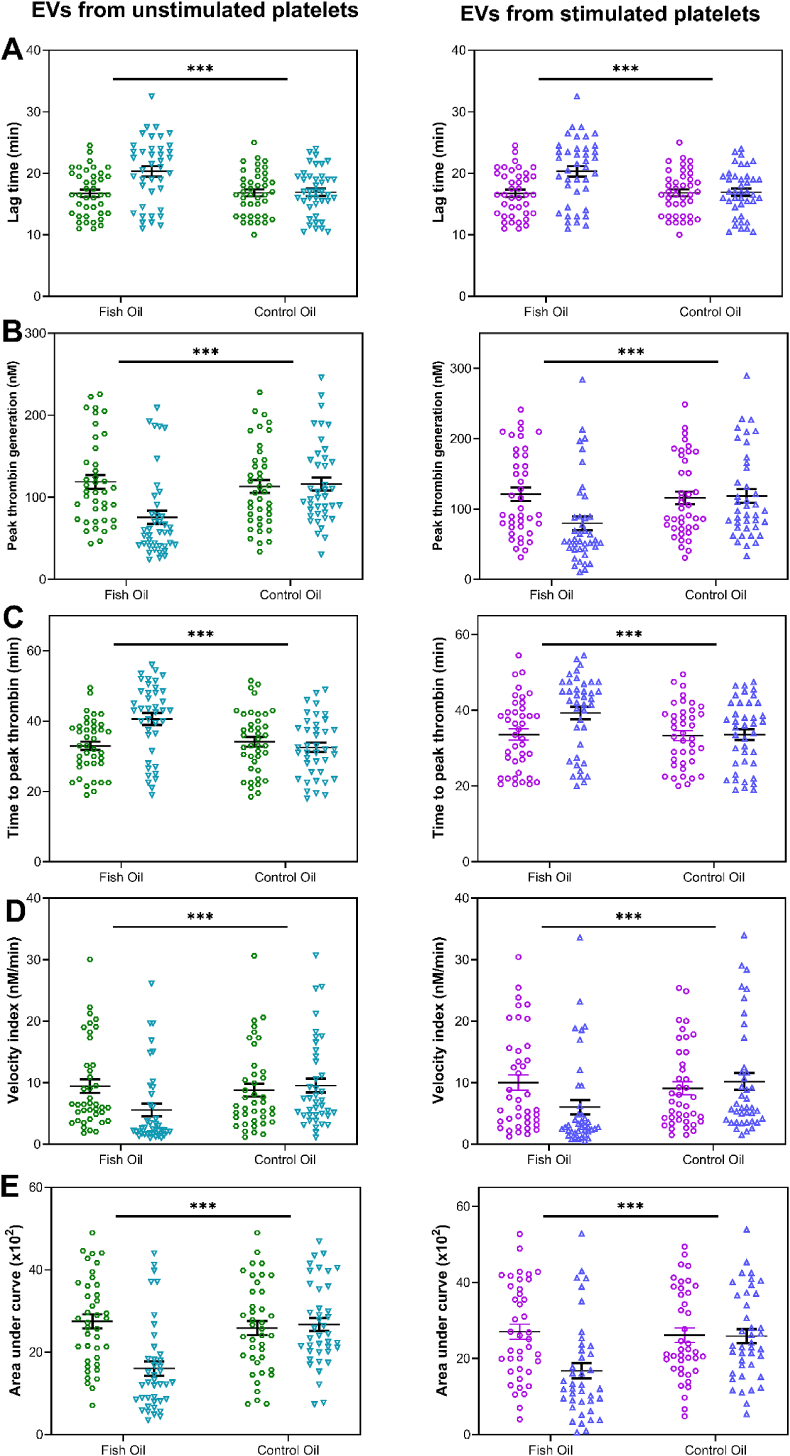
Thrombin generation induced by EVs generated in vitro from unstimulated or stimulated platelets before and after intervention. Circles represent baseline, and triangles represent after intervention. Data are mean ± SEM (n = 40). Comparisons after each intervention were drawn using the General Linear Model (GLM), including pairwise comparison with Bonferroni tests for treatment, period, and treatment∗time interaction, with differences shown at *P* < 0.05. Pooled VDP from healthy individuals (n = 3) was used as negative control. There was a significant effect of fish oil on (A) lag time for thrombin generation, (B) peak thrombin concentration, (C) time to reach peak thrombin, (D) velocity index, and (E) AUC for both (left panel) UP-EVs and (right panel) SP-EVs (treatment effects: *P* < 0.05; general linear model). *∗∗∗<* 0.001. EVs, extracellular vesicles; SP-EVs, stimulated platelet-derived extracellular vesicles; UP-EVs, unstimulated platelet-derived extracellular vesicles; VDP, vesicle-depleted plasma.

PDEVs derived from the stimulated/unstimulated platelets of participants supplemented with fish oil reduced fibrin clot formation and thrombin generation and increased fibrinolysis compared with those following the control intervention and for PDEVs derived from unstimulated platelets; there was also delayed clotting time (Supplemental Figures 4, 5 and Figure 5). However, there was no effect of the intervention on thrombus formation induced by PDEVs derived in vitro from stimulated platelets, including endpoints of thrombus formation, maximum thrombus formation, and AUC, although there was a trend for a decrease in these parameters (Supplemental Figure 6).

### Supplementation with fish oil altered the proteome of EVs generated in vitro from platelets

An untargeted approach to investigate global protein changes in the EV proteome following fish oil supplementation identified 409 proteins in EVs derived from stimulated platelets, of which 13 were exclusively present after fish oil and 42 only after control oil. For EVs derived from unstimulated platelets, a total of 595 proteins were identified, of which 33 were exclusively present after fish oil and 142 only after control oil (Supplemental Figure 7). Quantitative changes in proteins were expressed as fold change relative to the matched control sample, and analysis demonstrated a relative downregulation of proteins following fish oil supplementation. There were a total of 12 proteins in EVs derived from stimulated platelets, which had a log fold change > 1.5 after either fish oil or control oil, most of which were downregulated after fish oil (Supplemental Figure 8A). In contrast, these proteins were upregulated after control oil (Supplemental Figure 8B). The exception was WASH complex subunit 2A (WASHC2A), which was upregulated after both fish oil and control oil (Supplemental Figures 7, 8). For EVs from unstimulated platelets, there were a total of 21 proteins that demonstrated a log fold change >1.5 after either fish oil or control oil, and most were different from those altered in EVs from stimulated platelets. A total of 8 proteins were downregulated after fish oil, compared with little effect after control oil (Supplemental Figures 7, 8 C, D). However, 13 proteins were upregulated after fish oil but downregulated after control oil (Supplemental Figures 7, 8 C, D).

## Discussion

This study demonstrated that supplementing individuals with moderate risk of CVDs with fish oil altered the number and fatty acid profile of circulating EVs, enriching them with n-3 PUFAs and decreasing their capacity to support thrombin generation, as well as reducing the ability of EVs generated from n-3 PUFA-enriched platelets in vitro to support thrombin generation and clot formation. This raises the possibility that the anticoagulatory and cardioprotective properties of n-3 PUFAs could be mediated in a meaningful way through alterations in the number and function of circulating EVs. The study demonstrated wide-ranging effects of fish oil delivering 1.9 g/d n-3 PUFAs, which was sufficient to decrease blood pressure and plasma TAG concentration and alter EV number and ability to support thrombin generation. A significant reduction in plasma TAG concentration is a hallmark of fish oil supplementation [31] and the confirmatory results in this study therefore demonstrate the efficacy of the intervention and overall compliance of the subjects. The effect of n-3 PUFA supplementation on blood pressure is also broadly in agreement with published literature [32] and has been explained by an improvement in endothelial function in response to reduced systemic vascular resistance [33] and the vasodilatory effects of eicosanoids, whose metabolism is altered when synthesized from EPA and DHA [34]. The LDL-C raising effect of n-3 PUFAs has been known for some time [32] and is suggested to be due to an increase in particle size, mainly due to DHA, which reduces atherogenicity, and is therefore not necessarily detrimental [35]. In contrast, some of the high-profile trials suggest reduction of CVD events by EPA alone [36]. The relative effects of EPA compared with DHA on coagulation and fibrinolysis are poorly understood and the effects of both on platelet aggregation continue to be inconsistent, despite a large body of published data [37].

The current study is the first to assess the effect of n-3 PUFAs on fibrin clot properties using a spatial clot growth assay, providing a real-time observation of clot growth and lysis, and demonstrating significant effects on clot growth, but not on fibrinolysis. Some studies using high doses of n-3 PUFAs (ranging from 2 to 4 g/d) reported no effect of fish oil on fibrinolysis, [[38], [39], [40], [41]] whereas others reported an increase [42,43]. Fish oil does reduce levels of coagulation factors, including factors II, V, VII, and X and fibrinogen, which is consistent with the effects on clot growth [44].

The current study also demonstrated that supplementation with n-3 PUFAs reduced thrombin generation in PFP, in agreement with other well-controlled studies in healthy individuals [42] and patients with CAD [43] and diabetes mellitus [44]. Subjects at moderate risk for CVD demonstrated greater thrombin generation than healthy individuals and supplementation with n-3 PUFAs reduced thrombin generation to below the level seen in healthy individuals. An elevated level of thrombin generation has been associated with venous thromboembolism [44], and acute ischemic stroke [45], indicating that greater thrombin generation might be expected in people at risk for CVD. The assessment of thrombin generation in VDP provided insight into the impact of removal of EVs on thrombin generation, consistent with evidence that the presence of EVs in plasma enhanced tissue factor-stimulated thrombin generation [9]. Although TF and PS are thought to be the main contributors of the thrombogenicity of EVs, bioactive lipids, protein disulphide isomerase and factors VIII and Va have also been implicated [9].

Published data are broadly consistent with n-3 PUFAs decreasing numbers of circulating EVs and/or EV subpopulations, although studies have tended to be small, uncontrolled, included people with wide-ranging characteristics, used a wide range of doses of n-3 PUFAs, did not account for confounding factors, such as age and BMI, and provided insufficient information about the collection, isolation and characterization of EVs [[18], [19], [20], [21], [22], [23], [24], [25], [26], [27]]. Although the exact mechanism by which n-3 PUFA intervention leads to a reduction in the number of circulating EVs and EV subpopulations is not fully understood, the incorporation of n-3 PUFAs into the phospholipids of cell membranes in response to fish oil supplementation may significantly influence the remodeling of membrane lipid and subsequently the generation and behavior of EVs [46,47]. Flaxseed oil-derived α-linolenic acid (ALA) has been shown to inhibit the process of externalizing PS to the outer leaflet of the cell membrane during cell activation, which is a critical stage in the production of EV [48]. Additionally, the release of EVs relies heavily on cholesterol, which is both plentiful and essential in the structure of membrane lipid rafts. DHA has been observed to modify the size and composition of these rafts, forming a distinct, DHA-rich, and highly disordered nonraft area because it resists separating from cholesterol [49]. Consequently, n-3 PUFAs, especially DHA, might disrupt lipid rafts to such an extent that it reduces the process of EV shedding.

Alteration of the plasma fatty acid profile and enrichment of n-3 PUFAs at the expense of n-6 PUFAs is often used as a marker of compliance in human intervention trials, as was demonstrated for plasma phosphatidylcholine (PC) and phosphatidylethanolamine (PE) in the current study. However, this has never previously been reported for EVs taken either directly or prepared from platelets from participants undergoing a fish oil intervention, where enrichment of EV phospholipids with n-3 PUFAs occurred at the expense of n-6 PUFAs, particularly AA, and sometimes also the monounsaturated fatty acid, oleic acid [50]. The remodeling of EV lipids is, therefore, highly novel and the doubling of EPA and DHA content in circulating EVs at the expense of oleic acid and AA is remarkable. Although fatty acid profile changes in EVs generated from platelets in vitro followed the same general pattern as for circulating EVs, it was surprising to see that only the EPA content (and not DHA content) was significantly higher in the EVs generated from platelets prepared following fish oil intervention. Therefore, there may be some selectivity with respect to incorporation of n-3 PUFAs into PDEVs generated in vitro; however, this requires further investigation. In both the circulating and in vitro-generated PDEVs, n-3 PUFAs replaced AA, which could be key to biological and functional consequences.

Preliminary observations using an untargeted proteomics apparoch suggested that fish oil altered the proteome of in vitro-generated PDEVs, regardless of whether they were derived from unstimulated or stimulated platelets, leading to changes in the expression of various proteins involved in the pathogenesis of CVDs. However, although the expression of some proinflammatory and/or proatherosclerotic proteins, such as RBP4, PF4V1, ESAM and FBLN1, was downregulated and the nature of the stimulus for EV generation by platelets appeared to influence the proteome profile, a clear pattern was lacking. It is also important to consider that EVs are a rich source of bioactive lipids and analysis of the lipidome would provide a much fuller understanding of the impact of n-3 PUFA.

Supplementation with n-3 PUFAs significantly reduced the thrombogenicity of both circulating EVs and in vitro-generated PDEVs, regardless of whether they were derived from unstimulated or stimulated platelets, but did not alter thrombus formation in a whole blood assay, despite the marked effects on clot formation and thrombin generation, most likely due to the practicalities of generating sufficient numbers of PDEVs to employ in an ex vivo thrombus formation assay requiring continuous flow of particles. Overcoming these technical limitations would be key to addressing the question of the biological and clinical implications of the observations reported in this article and a lack of effect, therefore, should, not be taken to downplay the effects of n-3 PUFA on EV thrombogenicity.

Thrombin generation has been shown to be associated with phospholipid concentration in the plasma [51] and surface exposure of PS, as well as TF, on EVs is key to thrombin generation as they promote the assembly and activation of the prothrombinase complex to form thrombin [11]. The fact that n-3 PUFAs decreased PS expression by PDEVs in the current study may therefore at least partially account for the antithrombogenic effects of fish oil on thrombin generation induced by PDEVs. Replacement of AA with EPA in PDEVs produced in vitro from n-3 PUFA-enriched platelets may also affect thrombin generation through alterations in eicosanoid biosynthesis [52]. Although supplementation with fish oil also had favorable effects on both fibrin clot formation and on fibrinolysis triggered by PDEVs, there are no directly comparable published data, apart from the observation that supplementation post myocardial infarction with 5.2 g/d of n-3 PUFAs for 12 wk reduced fibrin generation capacity by prolonging lag time to clotting, and this was associated with a reduction in CD61+ PDEV numbers [23]. Thus, n-3 PUFAs could potentially alter the thrombogenicity of EVs through multiple mechanisms: a reduction in the number of circulating EVs, reduced expression of TF and/or PS, or alterations in bioactive lipids or other bioactive cargo.

The strengths of this study include the fact that it has a suitably powered crossover design with an appropriate length of intervention and washout period, and it demonstrates significant changes in the fatty acid composition of both plasma phospholipids and EVs, with corresponding dramatic effects on EV-dependent thrombin generation, the main outcome of the study. The main limitations of the study relate to the general lack of standardization of EV isolation and analysis, the challenges associated with characterizing a heterogeneous population of small particles, and the untargeted nature of the proteomics analysis. Future work will focus on mechanisms by which modification of the fatty acid composition of EVs alters thrombin generation.

In conclusion, n-3 PUFAs decreased numbers of circulating EVs, altered their fatty acid and proteome profile, and reduced their procoagulant activity in participants at moderate risk of CVDs. EVs derived in vitro from n-3 PUFA-enriched platelets also demonstrated reduced coagulatory activity, but did not alter thrombus formation in a whole blood ex vivo assay. Therefore, this study, provides novel evidence of potential anticoagulatory activity of n-3 PUFAs mediated through modification of EVs.

## Acknowledgments

Wiley Companies, manufacturer of AlaskOmega omega-3 concentrates, provided both the control and the fish oil capsules used in this trial.

## Author contributions

The authors’ responsibilities were as follows – PY is principle investigator and had overall responsibility for the design and supervision of the trial. PY, JG, CJ: secured funding. EB, RZ: were responsible for all operational elements of the trial and the majority of the analysis of samples, with input from SS, KA-R, JLM, HLF, SH, and RF under the supervision of PY, JG, CJ, and PCC. EB, RZ, JLM, SH, RF, PY: conducted data analysis. EB, RZ, PY: wrote the manuscript. PY is responsible for the final content; and all authors: read and approved the final version of the manuscript.

## Conflict of interest

The authors report no conflicts of interest.

## Funding

This research was funded by a grant to PY, JG, and CJ from the Biotechnology and Biological Sciences Research Council, United Kingdom (reference BB/N021185/1).

## Data availability

Data described in the manuscript will be made publicly and freely available without restriction from the University of Reading Research Data Archive at [URL: https://doi.org/10.17864/1947.000513]
