# Supplementary material for: Dietary n-3 polyunsaturated fatty acids alter the number, fatty acid profile and coagulatory activity of circulating and platelet-derived extracellular vesicles: a randomized, controlled crossover trial
Source: Am J Clin Nutr. 2024 Mar 13;119(5):1175–86. doi: 10.1016/j.ajcnut.2024.03.008 (PMC11130656; doi:10.1016/j.ajcnut.2024.03.008)
Supplement: Multimedia component 1 [file mmc1.docx]

**Dietary n-3 polyunsaturated fatty acids alter the number, fatty acid profile and coagulatory activity of circulating and platelet-derived extracellular vesicles: a randomized, controlled crossover trial**

Bozbas, E et al.

**Online Supplementary Material**

# **Supplementary Methods**

## **Functional assays of PDEVs**

### ***Measurement of clot formation induced by PDEVs***

The assay was set up by combining VDP (30% volume) in clot buffer (10 mM Tris [pH 7.4] and 0.01% Tween 20) with 5 µg/ml of PDEVs (final protein concentration) in 96-well flat-bottom microplates (Greiner Bio-One, Stonehouse, Gloucestershire, UK). Fibrin clot formation was then initiated by addition of 5 mM CaCl_2_ in a final volume of 100 µl of clotting mixture. Plates were read to determine a change in turbidity of the VDP at 37 °C for 1 hour at 30 second intervals using a fluorescence plate reader (FlexStation 3, United State) at a wavelength of 405 nm. Data were then analysed by an online application (https://drclongstaff.shinyapps.io/Clot_or_HaloCL/) to obtain relative parameters: time to 50% change, absorbance at 50% change, absorbance at peak and area under the curve (AUC).

### ***Measurement of fibrinolysis by PDEVs***

The assay for fibrinolysis of PDEVs generated in vitro was set up by combining VDP (30% final volume) in clot buffer (10 mM Tris [pH 7.4] and 0.01% Tween 20) with tPA (100 pM; Sigma-Aldrich, Dorset, UK) and 5 µg/ml PDEVs (final protein concentration) in 96-well flat-bottom microplates (Greiner Bio-One, Stonehouse, Gloucestershire, UK). Fibrin clot formation to lysis was initiated by addition of 5 mM CaCl_2_ in a final volume of 100 µl of clotting mixture. Plates were read at 37 °C for 8 hours at 30 second intervals using a fluorescence plate reader (FlexStation 3, United State) at a wavelength of 405 nm. Data were then analysed by an online application (https://drclongstaff.shinyapps.io/Clot_or_HaloCL/) to obtain relative parameters: area under the curve, time to 50% lysis from zero, absorbance at 50% lysis, time from 50% clotting to lysis and time between peak clotting and 50% lysis.

### ***Measurement of ex vivo thrombus formation by PDEVs under flow***

The assay was performed by adding either buffer (control) or 30 µl of PDEVs generated from TRAP-6 stimulated platelets (5 µg/ml final protein concentration of EVs) into whole blood in modified Tyrode’s-HEPES buffer (134 mM NaCl, 2.9 mM KCL, 0.34 mM Na2HPO4, 20 mM HEPES, 1 mM MgCl2, pH 7.4). Cellix Vena8 Fluoro+ biochips with capillaries 0.01 cm high and 0.04 cm wide were coated with collagen (100 mg/ml) prior to thrombus formation studies. Whole citrated blood labelled with 3,3'-Dihexyloxacarbocyanine Iodide (DiOC6) was allowed to flow for 10 minutes over the collagen coated capillary chambers to form platelet thrombi at an arterial shear rate of 1000s-1. Images were taken every 2-4 seconds using a Nikon A1R fluorescence confocal microscope at 20x magnification. Data were analysed using Fiji (Image J) by measuring the fluorescence intensity of the DiOC6 over time which corresponded to the increase in thrombus size.

## **Fatty acid composition and lipid profile analysis**

The fatty acid composition of EVs and plasma phospholipids was analysed as previously described (2). Briefly, an 800 µl aliquot of circulating EVs (isolated by SEC) was subjected to total lipid extraction For PDEV samples, 0.9% NaCl was added to adjust to 800 µl. Lipid was extracted from circulating EVs and PDEVs using chloroform and methanol in a 2:1 (v/v) ratio in the presence of butylated hydroxytoluene (50 mg/l). Sodium chloride was added to aid the separation into aqueous and organic lipid containing phases. The mixture was centrifuged at 1,000 x g for 10 min, low brake, at room temperature. The lower phase was collected into a new glass tube and dried under nitrogen at 40 °C. The lipid extracts were then dissolved in methanol containing 2% (v/v) sulphuric acid and incubated at 50°C for 2 h to generate fatty acid methyl esters (FAME). FAME were extracted using hexane and separated by gas chromatography using an Agilent 6890 series gas chromatograph equipped with flame ionisation detection (Agilent Technologies) and a BPX-70 fused silica capillary column (30m×0·25mm×25 μm; SGE Analytical Science. Quantification (with the use of the internal standards) was performed using ChemStation software (Agilent Technologies) and Microsoft Excel (Microsoft Corporation) by Helena Fisk. Results were reported as percent of total fatty acids.

For fatty acid composition of plasma phospholipids, a 400µl aliquot of frozen PFP was defrosted at room temperature using a roller mixer and centrifuged at 13,000 x g for 5 minutes at room temperature to remove denatured protein. 400 µl of 0.9% NaCl was added to the PFP sample and 30µg of phosphatidylcholine (PC) and 15µg of phosphatidylethanolamine (PE) internal standards were then added for quantitative analysis. Next, lipid extraction of plasma was performed as for EVs (see above). After drying with nitrogen, the lipid extract was dissolved in 1.0ml dry chloroform and transferred into chloroform-washed (2 x 1ml under vacuum) solid-phase extraction cartridges for separation of phospholipids. PC and PE were eluted by the addition of 2.0ml dry chloroform: methanol (60:40, v/v) and 2.0ml dry methanol respectively under vacuum. The PC and PE extracts were dried under nitrogen at 40°C. Before analysis by GC, the methyl esterification of plasma phospholipid extracts was performed as for EVs (see above).

For lipid profile analysis of plasma, PFP was defrosted at room temperature using a roller mixer, centrifuged at 500 x g for 5 min at room temperature and analysed for total cholesterol (TC), TAG, high density lipoprotein-cholesterol (HDL-C), low density lipoprotein-cholesterol (LDL-C) and TC/HDL-C ratio by a RANDOX clinical analyser (RANDOX Daytona+ Analyser, Randox Laboratories Ltd, United Kingdom).

## **Proteomics**

EV suspensions containing 50 µg protein were digested using FASP digestion (Vacacon500, Sartorius, VN01H02 10kDA), and sample volumes were adjusted to 100 µL with 8 M urea. The proteins were denatured with 100 µL of 8 M urea in 100 mM triethylammonium bicarbonate (TEAB) for 30 minutes at room temperature. The samples were then reduced by the addition of Tris (2-carboxyethl)phosphine (TCEP) to a final concentration of 50 mM for 30 minutes at room temperature, and alkylated by adding carbonyl alkylative amination (CAA) to a final concentration of 50 mM for 30 minutes at room temperature in the dark. The filter device containing the mixture was centrifuged at 14,300 x g for 10 minutes at room temperature. Digestion was achieved with the addition of 2.5 µg of trypsin in 200 µL of 50 mM TEAB for overnight at 37 °C. Peptides were quenched in two-washes with 200 µL of 0.1% trifluoroacetic (TFA) and 200 µL of 50% acetonitrile (ACN) in 0.1% TFA, each spun through the filter to collect all remaining sample. Collected samples were then dried down in a Speedvac.  Dried peptides were reconstituted in 21 µL of 5% formic acid and 5% DMSO and were analysed by LC-MS/MS on a Fusion-Lumos (Thermo Fischer Scientific, UK). Data were acquired in Data-Independent Acquisition (DIA). A library-free data search was performed in DIA-NN 1.8.1 with the database UPR UPR_Homosapiens_9606_UP000005640_20200803.fasta. False discovery rate (FDR) was set to 0.01 and match between runs (MBR) was enabled. MS intensities were normalised by MaxLFQ (generic label free technology) and proteins were log(2) transformed.

# **Supplementary Tables**

| **Supplementary Table 1.**  **Dietary intake before and during the intervention** | | | | | | | |
| --- | --- | --- | --- | --- | --- | --- | --- |
|  | **Fish Oil** | | **Control Oil** | | ***p - value*** | | |
|  | Before | During | Before | During | Before-During Fish oil | Before-During Control oil | Before Fish oil-Before Control oil |
| Energy (kcal/d) | 1606.5±90.6 | 1642.2±87.3 | 1642.0±84.8 | 1569.5±74.7 | 0.480 | 0.056 | 0.399 |
| Carbohydrate (g/d) | 195.2±12.4 | 196.7±12.1 | 196.2±11.8 | 187.7±10.2 | 0.812 | 0.112 | 0.881 |
| Protein (g/d) | 70.3±3.6 | 73.7±4.1 | 74.9±3.4 | 70.6±3.3 | 0.227 | 0.067 | 0.064 |
| Fat (g/d) | 60.0±3.8 | 61.3±3.5 | 64.9±3.5 | 60.5±3.3 | 0.588 | 0.067 | 0.055 |
| n-3 PUFA (g/d) | 1.1±0.1 | 1.1±0.1 | 1.2±0.1 | 1.1±0.1 | 0.989 | 0.060 | 0.131 |
| Total PUFA (g/d) | 11.6±0.9 | 12.0±0.9 | 12.1±0.7 | 11.1±0.7 | 0.511 | 0.054 | 0.382 |
| Total MUFA (g/d) | 21.1±1.6 | 21.8±1.3 | 22.7±1.3 | 22.1±1.1 | 0.472 | 0.068 | 0.117 |
| Total SFA (g/d) | 22.9±1.3 | 21.5±1.2 | 21.9±1.3 | 20.6±1.2 | 0.498 | 0.116 | 0.163 |
| Alcohol (g/d) | 8.6±1.3 | 8.0±1.1 | 8.1±1.2 | 8.1±1.2 | 0.615 | 0.969 | 0.406 |
| Fibre (g/d) | 18.8±1.1 | 18.9±1.1 | 18.7±1.1 | 17.6±0.9 | 0.881 | 0.063 | 0.849 |
| Data are mean ± SEM. Retrospective dietary analysis was performed using a modified EPIC-Norfolk and FETA software at the baseline visit (before) and at the post-intervention visit (during). Differences in dietary intake were drawn using a paired t-test; MUFA, monounsaturated fatty acid; PUFA, polyunsaturated fatty acid; SFA; saturated fatty acid. | | | | | | | |

| **Supplementary Table 2. Fatty acid composition of plasma phospholipids before and after intervention** | | | | | | | | | | |
| --- | --- | --- | --- | --- | --- | --- | --- | --- | --- | --- |
|  | **Plasma total phospholipids (µg/ml)** | | | | | **Plasma total phospholipids (wt%)** | | | | |
|  | **Fish Oil** | | **Control Oil** | | ***p*-value** | **Fish Oil** | | **Control Oil** | | ***p*-value** |
|  | Before | After | Before | After | *treatment* | Before | After | Before | After | *treatment* |
| Palmitic acid (16:0) | 465.3±13.2 | 461.2±14.8 | 469.9±12.7 | 470.6±11.2 | NS | 29.5±0.2 | 29.7±0.2 | 29.5±0.2 | 29.2±0.2 | NS |
| Stearic acid (18:0) | 216.9±7.4 | 212.2±7.2 | 217.9±7.6 | 221.4±6.8 | NS | 14.0±0.2 | 13.9±0.2 | 13.9±0.1 | 14.0±0.1 | NS |
| Oleic acid (18:1n-9) | 179.4±6.4 | 164.6±7.0 | 185.8±6.3 | 186.9±7.0 | NS | 12.4±0.3 | 11.6±0.3 | 12.7±0.3 | 12.7±0.4 | NS |
| Vaccenic acid (18:1n-7) | 20.8±0.9 | 19.7±0.9 | 21.1±0.7 | 20.7±0.8 | NS | 1.3±0.03 | 1.3±0.02 | 1.4±0.04 | 1.3±0.04 | NS |
| Linoleic acid (18:2n-6) | 335.6±11.1 | 299.5±11.1 | 337.2±10.3 | 346.8±9.1 | <0.001 | 21.2±0.4 | 19.3±0.4 | 21.1±0.4 | 21.5±0.3 | <0.001 |
| ALA (18:3n-3) | 4.5±0.2 | 4.0±0.2 | 4.6±0.2 | 4.5±0.2 | NS | 0.3±0.01 | 0.3±0.02 | 0.3±0.01 | 0.3±0.01 | NS |
| DGLA (20:3n-6) | 49.4±2.2 | 35.9±2.0 | 48.8±2.3 | 49.6±2.1 | <0.001 | 3.1±0.1 | 2.2±0.1 | 3.0±0.1 | 3.0±0.1 | <0.001 |
| AA (20:4n-6) | 152.1±6.6 | 121.9±6.1 | 148.9±6.0 | 152.7±6.2 | <0.001 | 9.9±0.3 | 8.1±0.2 | 9.6±0.3 | 9.8±0.3 | <0.001 |
| ETA (20:4n-3) | 3.0±0.2 | 3.2±0.2 | 3.2±0.2 | 3.1±0.2 | NS | 0.2±0.01 | 0.3±0.01 | 0.2±0.01 | 0.2±0.01 | NS |
| EPA (20:5n-3) | 21.0±1.9 | 63.3±3.2 | 21.8±1.5 | 20.3±1.2 | <0.001 | 1.3±0.1 | 4.6±0.2 | 1.4±0.1 | 1.3±0.7 | <0.001 |
| DPA (22:5n-3) | 16.9±0.8 | 20.4±0.8 | 17.4±0.8 | 16.4±0.7 | <0.001 | 1.1±0.03 | 1.4±0.04 | 1.1±0.03 | 1.1±0.03 | <0.001 |
| DHA (22:6n-3) | 53.7±3.1 | 87.3±3.2 | 57.9±2.9 | 55.3±2.4 | <0.001 | 3.6±0.2 | 6.0±0.1 | 3.9±0.2 | 3.7±0.1 | <0.001 |
| Total SFAs | 682.3±20.0 | 673.4±21.2 | 687.9±19.8 | 692.0±17.5 | NS | 43.5±0.2 | 43.7±0.2 | 43.4±0.2 | 43.1±0.2 | NS |
| Total MUFAs | 215.9±7.4 | 198.1±8.5 | 223.2±7.5 | 223.3±8.3 | NS | 14.8±0.4 | 13.8±0.4 | 15.2±0.3 | 15.0±0.4 | NS |
| Total n-3 PUFAs | 99.1±5.1 | 178.3±6.6 | 104.8±4.6 | 99.4±3.8 | <0.001 | 6.6±0.2 | 12.2±0.3 | 6.8±0.2 | 6.6±0.7 | <0.001 |
| Total n-6 PUFAs | 537.0±16.7 | 457.4±16.7 | 534.9±15.1 | 549.2±13.9 | <0.001 | 34.2±0.3 | 29.6±0.5 | 33.7±0.3 | 34.3±0.3 | <0.001 |
| Data are mean ± SEM (n = 40). A 400µl aliquot of PFP was mixed with an equal volume of 0.9% NaCl and 30µg of phosphatidylcholine (PC) and 15µg of phosphatidylethanolamine (PE) internal standards. Lipid was extracted using chloroform and methanol in a 2:1 (v/v) ratio in the presence of butylated hydroxytoluene (50 mg/l), as described in the methods. Comparisons after each intervention were drawn using General Linear Model (GLM), with differences shown at p < 0.05. *AA, arachidonic acid; ALA, α-linolenic acid; DGLA, dihomo-γ-linolenic acid; DHA, docosahexaenoic acid; DPA, docosapentaenoic acid; EPA, eicosapentaenoic acid; ETA, eicosatetraenoic acid; MUFA, monounsaturated fatty acid; PUFA, polyunsaturated fatty acid; SFA, saturated fatty acid.* | | | | | | | | | | |

| **Supplementary Table 3. Fatty acid composition of plasma PC and PE before and after intervention** | | | | | | | | | | |
| --- | --- | --- | --- | --- | --- | --- | --- | --- | --- | --- |
|  | **PC** | | | | | **PE** | | | | |
|  | **Fish Oil** | | **Control Oil** | | ***p*-value** | **Fish Oil** | | **Control Oil** | | ***p*-value** |
|  | Before  (wt%) | After  (wt%) | Before  (wt%) | After  (wt%) | *treatment* | Before  (wt%) | After  (wt%) | Before  (wt%) | After  (wt%) | *treatment* |
| Myristic acid (14:0) | 0.4±0.0 | 0.4±0.0 | 0.4±0.0 | 0.4±0.0 | 0.207 | 0.9±0.1 | 0.9±0.0 | 0.9±0.0 | 0.9±0.0 | 0.986 |
| Palmitic acid (16:0) | 30.8±0.2 | 30.1±0.2 | 30.8±0.2 | 30.59±0.2 | 0.048 | 22.6±0.3 | 22.0±0.3 | 22.6±0.3 | 22.0±0.3 | 0.855 |
| Palmitoleic acid (16:1n-7) | 0.8±0.0 | 0.7±0.0 | 0.8±0.0 | 0.74±0.0 | 0.048 | 1.4±0.1 | 1.2±0.1 | 1.5±0.1 | 1.6±0.1 | 0.713 |
| Stearic acid (18:0) | 14.0±0.2 | 14.0±0.2 | 13.9±0.2 | 14.02±0.2 | 0.576 | 14.3±0.3 | 13.7±0.2 | 14.1±0.3 | 13.9±0.2 | 0.422 |
| Oleic acid (18:1n-9) | 10.8±0.2 | 10.1±0.2 | 10.9±0.2 | 10.99±0.2 | 0.004 | 20.0±1.1 | 18.2±1.1 | 20.9±1.1 | 19.6±1.1 | 0.755 |
| Vaccenic acid (18:1n-7) | 1.3±0.0 | 1.3±0.0 | 1.4±0.0 | 1.33±0.0 | 0.573 | 1.3±0.1 | 1.2±0.1 | 1.3±0.1 | 1.3±0.1 | 0.643 |
| Linoleic acid (18:2n-6) | 22.3±0.4 | 22.2±0.4 | 22.2±0.4 | 22.52±0.3 | <0.001 | 15.4±0.3 | 14.0±0.4 | 15.2±0.3 | 16.0±0.3 | <0.001 |
| GLA (18:3n-6) | 0.1±0.0 | 0.1±0.0 | 0.1±0.0 | 0.09±0.0 | <0.001 | 0.1±0.0 | 0.1±0.0 | 0.1±0.0 | 0.1±0.0 | 0.087 |
| ALA (18:3n-3) | 0.2±0.0 | 0.2±0.0 | 0.2±0.0 | 0.2±0.0 | 0.575 | 0.8±0.0 | 0.8±0.1 | 0.8±0.1 | 0.8±0.1 | 0.208 |
| Arachidic acid (20:0) | 0.1±0.0 | 0.1±0.0 | 0.1±0.0 | 0.1±0.0 | 0.697 | 0.2±0.01 | 0.2±0.0 | 0.2±0.0 | 0.2±0.0 | 0.723 |
| Eicosenoic acid (20:1n-9) | 0.2±0.0 | 0.2±0.0 | 0.2±0.0 | 0.2±0.0 | 0.188 | 0.4±0.0 | 0.3±0.0 | 0.3±0.0 | 0.3±0.0 | 0.232 |
| Eicosadienoic acid (20:2n-6) | 0.2±0.0 | 0.2±0.0 | 0.2±0.0 | 0.2±0.0 | <0.001 | 0.3±0.0 | 0.3±0.0 | 0.3±0.0 | 0.3±0.0 | 0.195 |
| DGLA (20:3n-6) | 3.3±0.1 | 2.4±0.1 | 3.2±0.1 | 3.3±0.1 | <0.001 | 1.8±0.1 | 1.3±0.1 | 1.6±0.10 | 1.7±0.1 | <0.001 |
| AA (20:4n-6) | 9.7±0.3 | 7.9±0.2 | 9.5±0.3 | 9.6±0.3 | <0.001 | 11.5±0.5 | 9.6±0.5 | 10.9±0.6 | 12.0±0.6 | 0.001 |
| ETA (20:4n-3) | 0.2±0.0 | 0.2±0.0 | 0.2±0.0 | 0.2±0.0 | 0.036 | 0.7±0.1 | 0.8±0.1 | 0.7±0.1 | 0.8±0.1 | 0.759 |
| EPA (20:5n-3) | 1.3±0.1 | 4.2±0.2 | 1.4±0.1 | 1.3±0.1 | <0.001 | 1.5±0.1 | 5.2±0.3 | 1.5±0.1 | 1.5±0.1 | <0.001 |
| DPA (22:5n-3) | 1.1±0.0 | 1.4±0.0 | 1.1±0.0 | 1.0±0.0 | <0.001 | 1.5±0.1 | 1.5±0.1 | 1.4±0.1 | 1.5±0.1 | 0.670 |
| DHA (22:6n-3) | 3.3±0.2 | 5.6±0.1 | 3.6±0.1 | 3.3±0.1 | <0.001 | 5.6±0.3 | 8.9±0.4 | 5.6±0.3 | 8.9±0.4 | <0.001 |
| Data are mean ± SEM (n = 40). Plasma lipid extracts were dissolved in 1.0 ml dry chloroform and transferred into chloroform-washed solid-phase extraction cartridges for separation of phospholipids. PC and PE were eluted by the addition of 2.0 ml dry chloroform: methanol (60:40, v/v) and 2.0 ml dry methanol respectively under vacuum, as described in the supplementary methods. Comparisons after each intervention were drawn using General Linear Model (GLM), with differences shown at p < 0.05. *AA, arachidonic acid; ALA, alpha-linolenic acid; DGLA, dihomo-γ-linolenic acid; ETA, eicosatetraenoic acid; EPA, eicosapentaenoic acid; DPA, docosapentaenoic acid; DHA, docosahexaenoic acid; GLA, gamma linolenic acid; PC, phosphatidylcholine; PE, phosphatidylethanolamine.* | | | | | | | | | | |

| **Supplementary Table 4. Traditional CVD risk markers before and after intervention** | | | | | | | |
| --- | --- | --- | --- | --- | --- | --- | --- |
|  | **Fish Oil** | | **Control Oil** | | ***p*-value** | | |
|  | Before | After | Before | After | *treatment* | *time* | *Treatment x time* |
| Systolic BP (mm Hg) | 134.03±2.18 | 127.38±2.19 | 131.85±2.19 | 135.23±2.14 | <0.001 | 0.011 | 0.033 |
| Diastolic BP (mm Hg) | 78.98±1.40 | 76.05±1.42 | 78.08±1.43 | 79.00±1.63 | 0.002 | 0.952 | 0.114 |
| BMI (kg/m^2^) | 25.57±0.48 | 25.62±0.48 | 25.47±0.46 | 25.52±0.47 | 0.675 | 0.076 | 0.675 |
| TAG (mmol/l) | 0.99±0.06 | 0.88±0.05 | 0.98±0.06 | 1.01±0.06 | 0.016 | 0.566 | 0.659 |
| Total-cholesterol (mmol/l) | 4.88±0.12 | 5.02±0.11 | 4.90±0.13 | 4.88±0.13 | 0.077 | 0.921 | 0.793 |
| HDL-cholesterol (mmol/l) | 1.39±0.05 | 1.45±0.05 | 1.38±0.05 | 1.42±0.05 | 0.379 | 0.938 | 0.341 |
| LDL-cholesterol (mmol/l) | 3.04±0.10 | 3.17±0.10 | 3.06±0.10 | 3.01±0.10 | 0.014 | 0.842 | 0.943 |
| Data are mean ± SEM, (n = 40). Comparisons after each intervention were drawn using the General Linear Model (GLM), with differences shown at p < 0.05. *BP, blood pressure; BMI, body mass index; TAG, triacylglycerol.* | | | | | | | |

| **Supplementary Table 5. Platelet aggregation parameters before and after intervention** | | | | | | |
| --- | --- | --- | --- | --- | --- | --- |
|  |  | **Fish Oil** | | **Control Oil** | | ***p-value*** |
| **Agonists** | **Parameters** | Before | After | Before | After | treatment |
| ADP | Maximum | 26.35 ± 1.75 | 27.23 ± 2.07 | 26.77 ± 2.00 | 23.70 ± 1.68 | *0.599* |
|  | Minimum | 90.30 ± 1.54 | 93.75 ± 1.85 | 90.64 ± 1.56 | 93.96 ± 1.35 | *0.204* |
|  | LogEC50 | -2.87 ± 0.03 | -2.94 ±0.03 | -2.89 ± 0.04 | -2.86 ± 0.05 | *0.336* |
|  | Hillslope | 1.46 ± 0.20 | 1.34 ± 0.20 | 1.33 ± 0.18 | 1.31 ± 0.14 | *0.935* |
| CRP-XL | Maximum | 25.33 ± 2.06 | 24.70 ± 2.48 | 27.14 ± 2.34 | 22.09 ± 2.33 | *0.515* |
|  | Minimum | 93.69 ± 1.39 | 94.07 ± 1.62 | 93.04 ± 1.39 | 93.83 ± 1.30 | *0.960* |
|  | LogEC50 | -4.64 ± 0.03 | -4.64 ± 0.03 | -4.75 ± 0.04 | -4.77 ± 0.05 | *0.061* |
|  | Hillslope | 1.50 ± 0.20 | 1.35 ± 0.19 | 1.51 ± 0.21 | 1.38 ± 0.17 | *0.897* |
| Epinephrine | Maximum | 23.84 ± 4.83 | 22.56 ± 4.86 | 26.38 ± 6.33 | 18.54 ±6.05 | *0.823* |
|  | Minimum | 84.19 ± 2.89 | 80.39 ± 3.20 | 85.88 ± 3.92 | 87.20 ± 3.11 | *0.575* |
|  | LogEC50 | -4.11 ± 0.12 | -4.04 ± 0.13 | -4.06 ± 0.16 | -4.18 ± 0.13 | *0.871* |
|  | Hillslope | 0.94 ± 0.23 | 0.92 ± 0.24 | 0.81 ± 0.25 | 0.82 ± 0.19 | *0.970* |
| TRAP-6 | Maximum | 12.46 ± 2.01 | 10.61 ± 2.05 | 16.89 ± 2.09 | 10.43 ± 1.81 | *0.109* |
|  | Minimum | 97.58 ± 4.38 | 93.36 ± 4.26 | 96.97 ± 5.39 | 92.00 ± 3.29 | *0.705* |
|  | LogEC50 | -2.46 ± 0.06 | -2.46 ± 0.06 | -2.33 ± 0.08 | -2.51 ± 0.05 | 0.187 |
|  | Hillslope | 1.20 ± 0.19 | 1.27 ± 0.21 | 1.25 ± 0.24 | 1.47 ± 0.22 | *0.815* |
| U46619 | Maximum | 18.92 ± 2.46 | 14.92 ± 2.49 | 19.30 ± 2.94 | 14.39 ± 2.94 | *0.439* |
|  | Minimum | 91.64 ± 1.79 | 90.87 ± 1.86 | 91.73 ± 2.05 | 93.05 ± 1.83 | *0.872* |
|  | LogEC50 | -3.59 ± 0.06 | -3.67± 0.05 | -3.59 ± 0.05 | -3.55 ± 0.05 | *0.312* |
|  | Hillslope | 1.48 ± 0.23 | 1.46 ± 0.22 | 1.36 ± 0.23 | 1.33 ± 0.19 | *0.948* |
| Data are mean ± SEM (n = 40). A high throughput, plate-based, platelet aggregation was conducted, as described in the main Methods. Maximum response, minimum response, Log EC50 and hill slope values were calculated from dose-response curves of platelet responses to ADP, CRP-XL, epinephrine, TRAP-6 and U46619. Comparisons after each intervention were drawn using 2-way ANOVA with the Turkey multiple comparisons test, with differences shown at p < 0.05. *ADP, adenosine diphosphate; TRAP-6, thrombin receptor activator peptide; CRP-XL, collagen-related peptides- cross-linking.* | | | | | | |

# **Supplementary Figures**

**
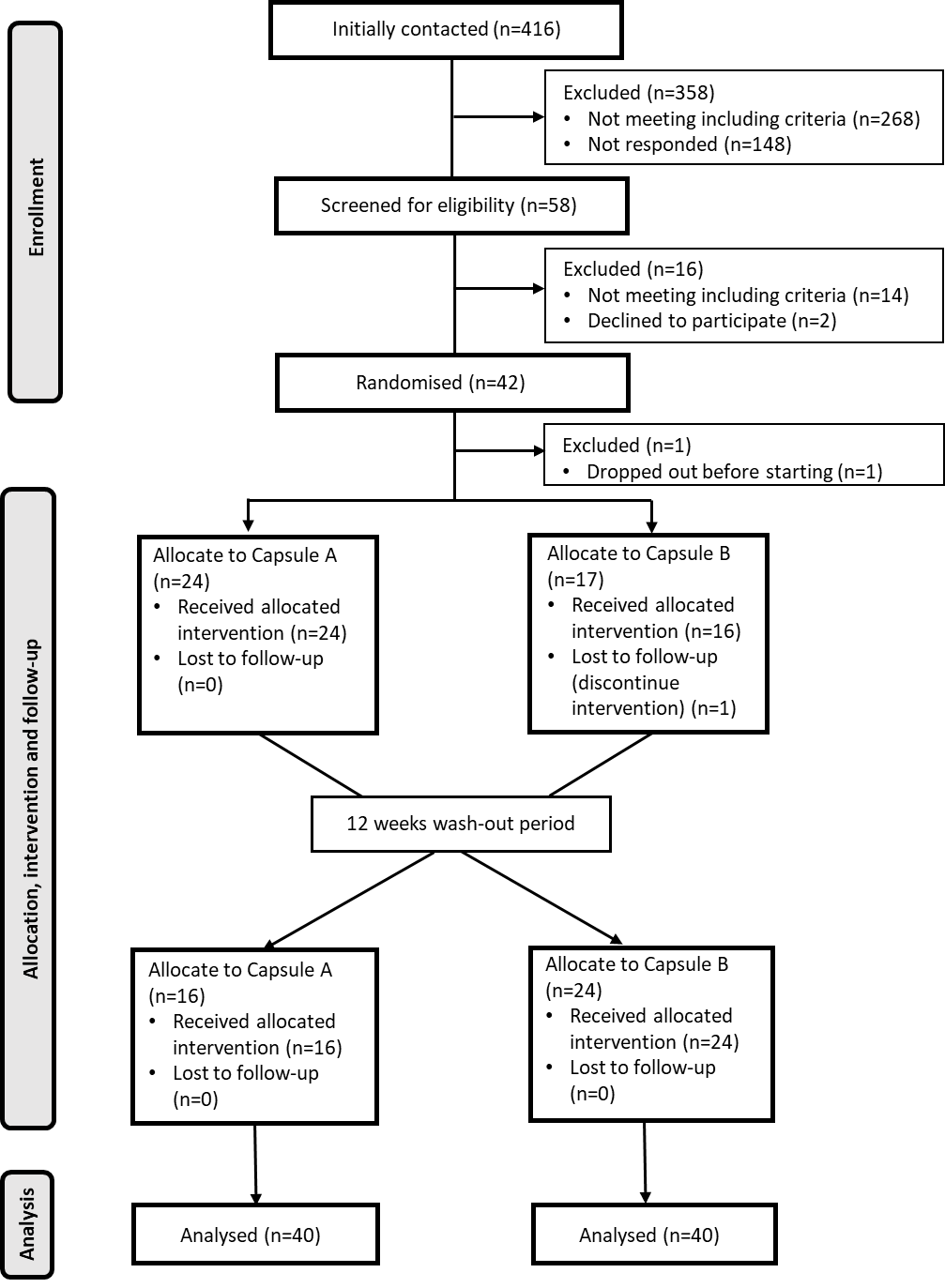
**

**Supplementary Figure 1. Participant flow.** Capsule A, control oil supplement; Capsule B, fish oil supplement.

 **Supplementary Figure 2. Removal of EVs during each stage of the preparation of VDP.** Venous blood samples were collected from three healthy, fasted participants to prepare pooled vesicle-depleted plasma (VDP). Whole blood was centrifuged twice at 2,500 x g for 15 min to remove blood cells (Plasma 1 and Plasma 2 respectively) and vesicles were depleted from the pooled plasma by ultracentrifugation at 20,000 x g for 1 hour at 4 °C, which pellets the EVs, leaving vesicle-poor supernatant (Plasma 3). The vesicle poor supernatant was ultracentrifuged at 100,000 x g for 1 hour at 4 °C (Plasma 4), followed by filtration four times through a 0.1 µm filter (Plasma 5 after 1^st^ filtration and VDF after 4^th^ filtration). Removal of vesicles at each stage of preparation of VDP was verified by NTA and FCM (Annexin V staining), which showed that 86.4% of particles detectable by NTA were removed from plasma (A), with 97.3% removal of PS+ EVs from plasma (B). *EVs, extracellular vesicles; PS+ EV, phosphatidylserine positive extracellular vesicles; VDP, vesicle-depleted plasma.*


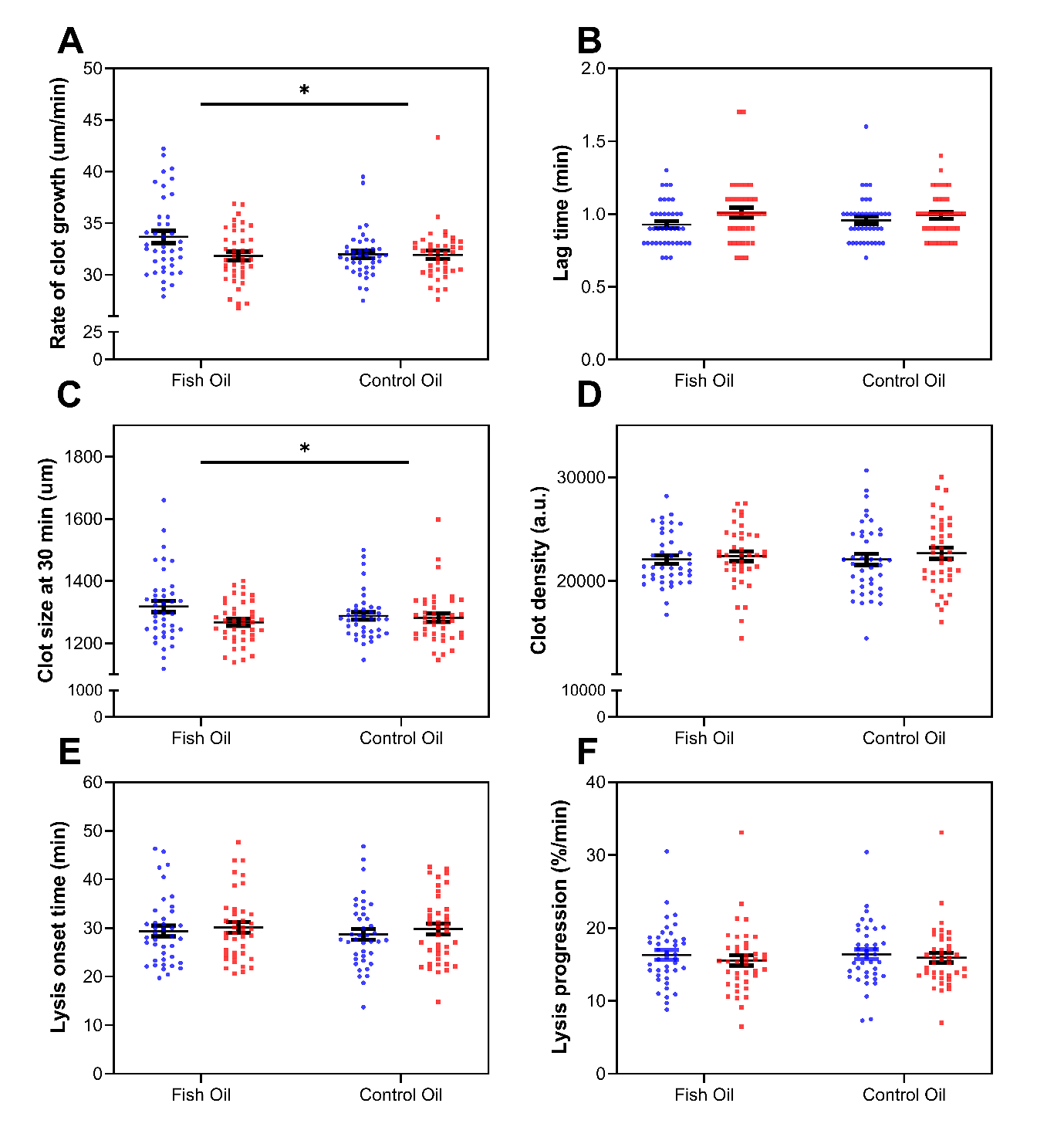
**Supplementary Figure 3. Coagulation and fibrinolysis parameters before and after intervention.** Thrombodynamics analysis was used to determine the formation and lysis of a fibrin clot, as described in the main methods. Blue circles represent before intervention and red circles represent after intervention. Data are mean ± SEM, (n=40). Comparisons after each intervention were drawn using General Linear Model (GLM), including pairwise comparison test with Bonferroni for treatment, period and treatment*time interaction, with differences shown at p < 0.05. *p<0.


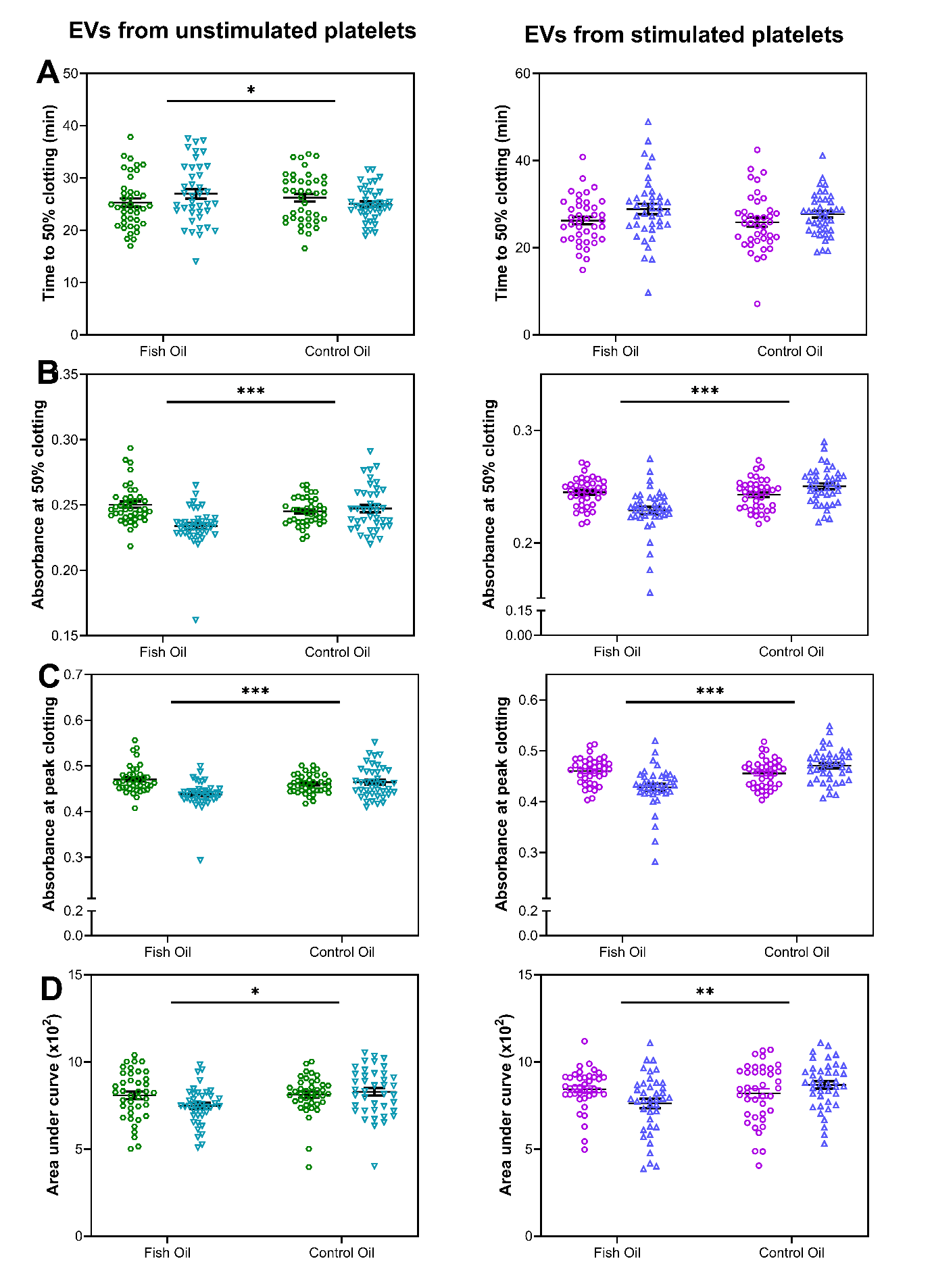
 **Supplementary Figure 4. Clot formation induced by EVs generated in vitro from unstimulated or stimulated platelets before and after intervention**. Thrombodynamics analysis was used to determine the formation of a fibrin clot, as described in the supplementary methods. Circles represent baseline and triangles represent after intervention. Data are mean ± SEM, (n=40). Comparisons after each intervention were made using the General Linear Model (GLM), including pairwise comparison with Bonferroni test for treatment, period and treatment*time interaction, with differences shown at p < 0.05. Pooled VDP from healthy subjects (n=3) was served as negative control. There was significant effect of fish oil on **(A left panel)** time to 50% clotting for only UP-EVs, while there was significant effect of fish oil on **(B)** absorbance at 50% clotting, **(C)** absorbance at peak and **(D)** area under curve for both (**left panel**) UP-EVs and (**right panel**) SP-EVs (treatment effects: p < 0.05; general linear model). There was no significant effect of time and no treatment*time interaction with respect to any parameter (overall effects: p > 0.05; general linear model), apart from a significant effect of treatment*time interaction with respect absorbance at 50% clotting and absorbance at peak for UP-EV (treatment*time effects: p < 0.05; general linear model). **p<0.05, **p<0.01 and ***p<0.001*. *SP-EVs, stimulated platelet-derived extracellular vesicles; UP-EVs, unstimulated platelet-derived extracellular vesicles; VDP, vesicle-depleted plasma.*


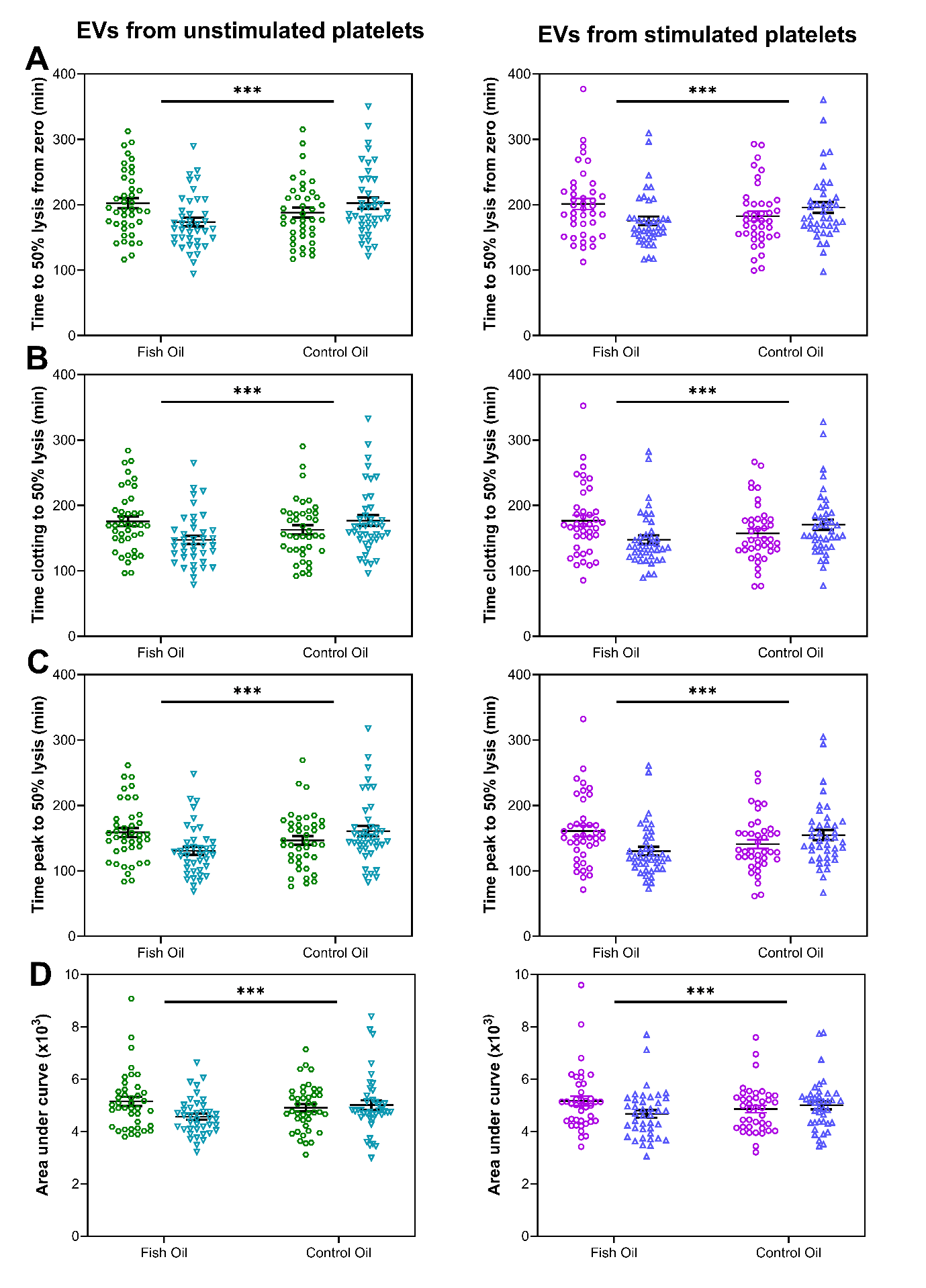
 **Supplementary Figure 5. Fibrinolysis induced by EVs generated in vitro from unstimulated or stimulated platelets before and after intervention**. Thrombodynamics analysis was used to determine the lysis of a fibrin clot, as described in the supplementary methods. Circles represent baseline and triangles represent after intervention.. Data are mean ± SEM, (n=40). Comparisons after each intervention were made using the General Linear Model (GLM), including pairwise comparison with Bonferroni test for treatment, period and treatment*time interaction, with differences shown at p < 0.05. Pooled VDP from healthy subjects (n=3) was served as negative control. There was significant effect of fish oil on **(A)** time to 50% lysis from zero, **(B)** time clotting to 50% lysis, **(C)** time peak to 50% lysis and **(D)** area under curve for both UP-EVs (**left panel**) and SP-EVs (**right panel**) (treatment effects: p < 0.05; general linear model). *SP-EVs, stimulated platelet-derived extracellular vesicles; UP-EVs, unstimulated platelet-derived extracellular vesicles; VDP, vesicle-depleted plasma.*


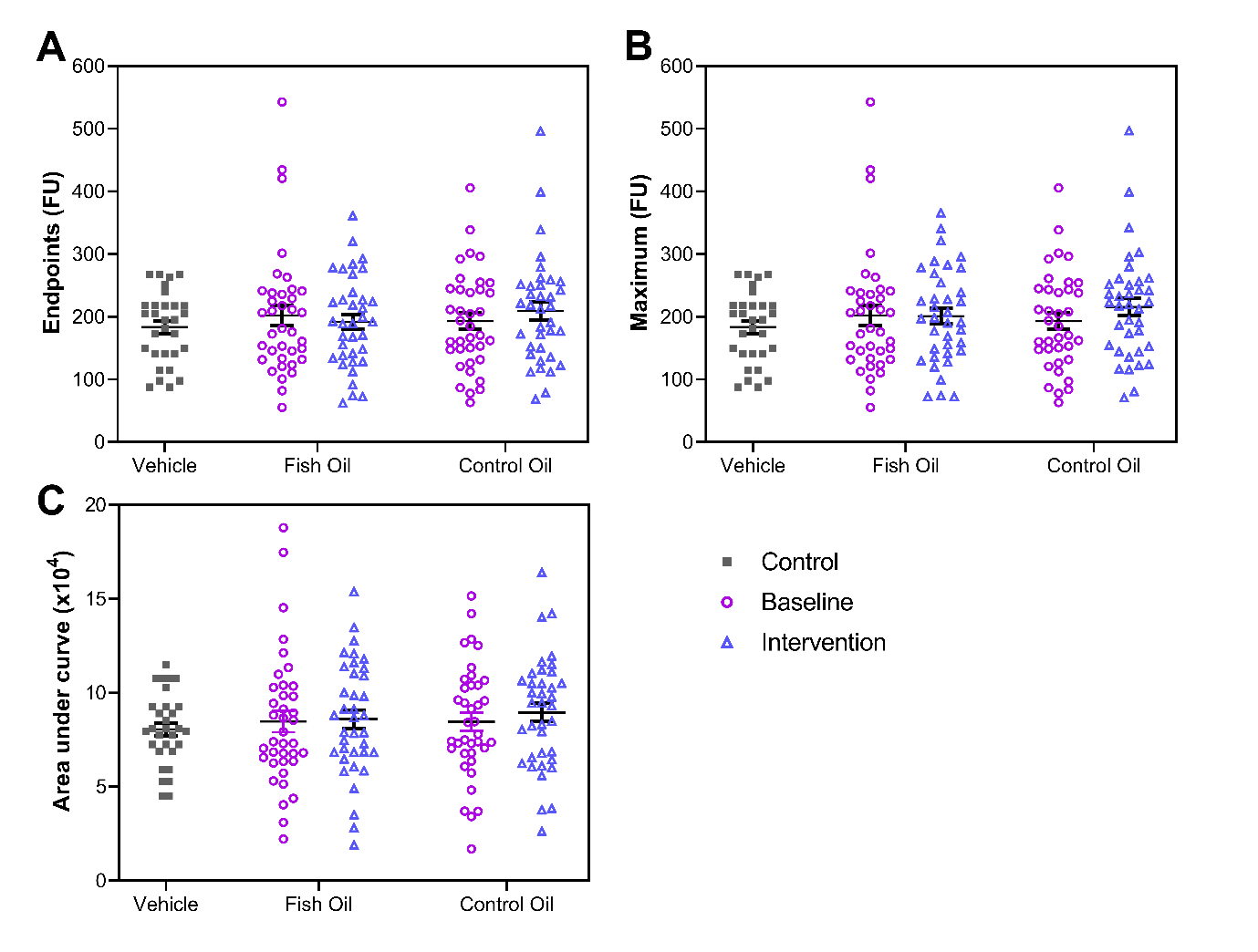
 **Supplementary Figure 6. Thrombus formation in the presence of PDEVs derived from n-3 PUFA-enriched stimulated platelets**. Thrombus formation was assessed in a whole blood system using collagen coated capillary chambers to form platelet thrombi at an arterial shear rate of 1000s-1 is response to addition of PDEVs, as described in the supplementary methods. Images were taken every 2-4 seconds using a Nikon A1R fluorescence confocal microscope at 20x magnification. Fluorescence intensity of the DiOC6 over time corresponded to the increase in thrombus size. Data are mean ± SEM, (n=40). Circles represent baseline and triangles represent after intervention samples. Comparisons after each intervention were made using General Linear Model (GLM), including pairwise comparison test with Bonferroni for treatment, period and treatment*time interaction, with differences shown at p < 0.05. There was no significant effect of interventions on **(A)** endpoints of thrombus formation, **(B)** maximal thrombus formation and **(C)** area under curve (treatment effect: p > 0.05; general linear model).


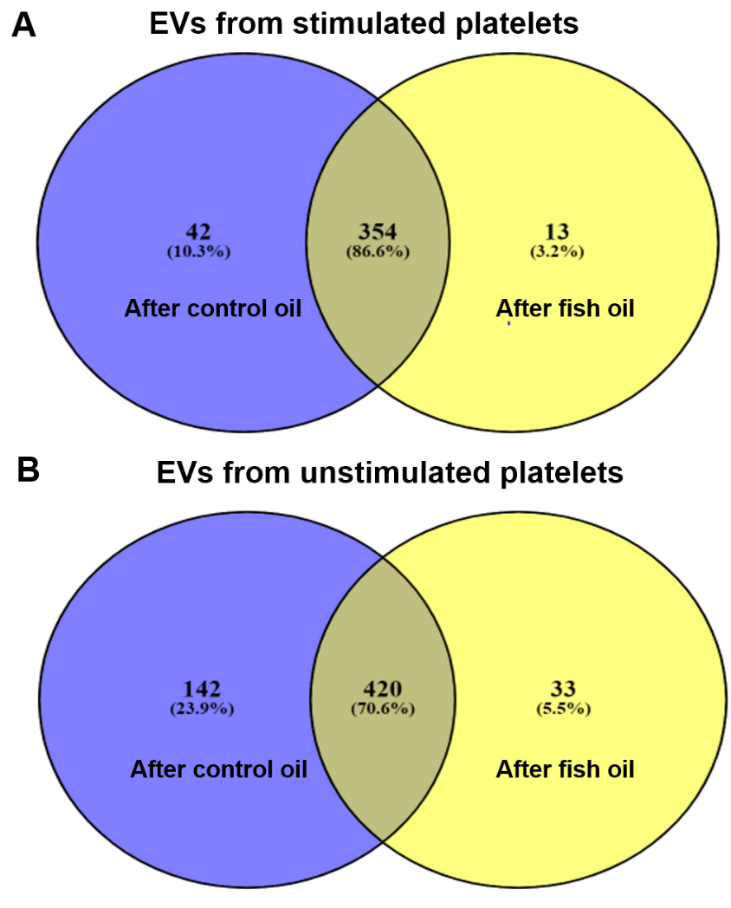


**Supplementary Figure 7. Proteomic profiling of EVs generated *in vitro* from platelets.** EV suspensions containing 50 µg protein were subjected to proteomics analysis, as described in the supplementary methods. A library-free data search was performed in DIA-NN 1.8.1 with the database UPR UPR_Homosapiens_9606_UP000005640_20200803.fasta. False discovery rate (FDR) was set to 0.01 and match between runs (MBR) was enabled. MS intensities were normalised by MaxLFQ (generic label free technology) and proteins were log(2) transformed.

The Venn diagram reports the numbers of proteins identified (**A**) in the EVs derived from stimulated platelets samples after control oil and after fish oil intervention, and (**B**) in the EVs derived from unstimulated platelets samples after control oil and after fish oil intervention.


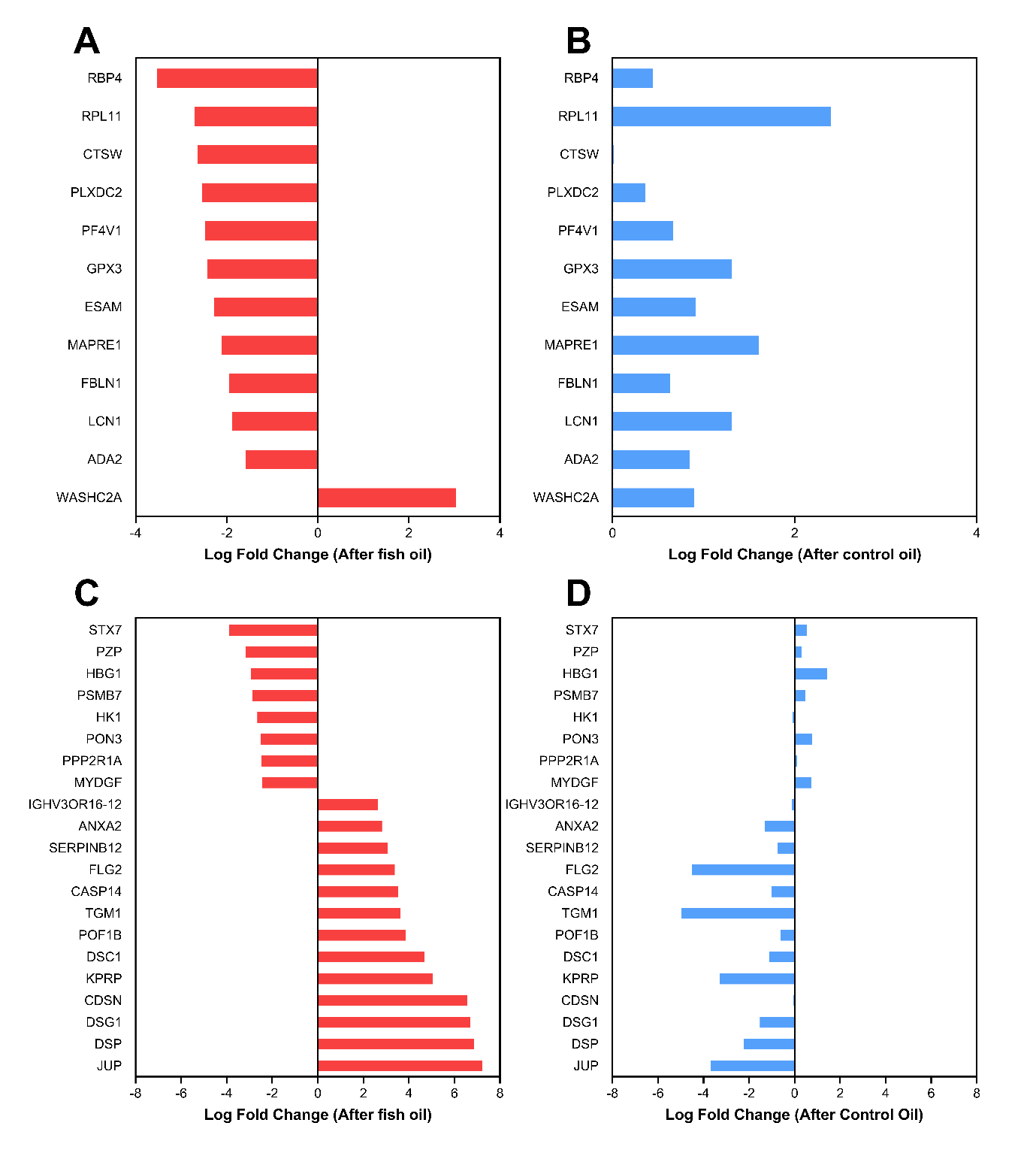
 **Supplementary Figure 8. Proteome analysis of EVs generated *in vitro* from stimulated or unstimulated platelets before and after intervention**. EV suspensions containing 50 µg protein were subjected to proteomics analysis, as described in the supplementary methods. A library-free data search was performed in DIA-NN 1.8.1 with the database UPR UPR_Homosapiens_9606_UP000005640_20200803.fasta. False discovery rate (FDR) was set to 0.01 and match between runs (MBR) was enabled. MS intensities were normalised by MaxLFQ (generic label free technology) and proteins were log(2) transformed. The Log Fold Change indicates the magnitude of change in atherosclerosis and CVD related proteins in EVs generated from stimulated platelets after either fish oil (**A**) or control oil (**B**) compared to baseline, and in EVs generated from unstimulated platelets after either fish oil (**C**) or control oil (**D**) compared to baseline. All changes were *p < 0.05.*

***References***

1. Mulligan AA, Luben RN, Bhaniani A, Parry-Smith DJ, O'Connor L, Khawaja AP, Forouhi NG, Khaw K-T. A new tool for converting food frequency questionnaire data into nutrient and food group values: FETA research methods and availability. BMJ open. 2014;4(3).

2. Fisk HL, West AL, Childs CE, Burdge GC, Calder PC. The use of gas chromatography to analyze compositional changes of fatty acids in rat liver tissue during pregnancy. Journal of visualized experiments: JoVE. 2014(85).
